# Crosstalk between the Protein Surface and Hydrophobic Core in a Core-swapped Fibronectin Type III Domain

**DOI:** 10.1016/j.jmb.2007.10.056

**Published:** 2008-01-11

**Authors:** Kate S. Billings, Robert B. Best, Trevor J. Rutherford, Jane Clarke

**Affiliations:** 1 Cambridge University Chemical Laboratory, MRC Centre for Protein Engineering, Lensfield Road, Cambridge CB2 1EW, UK; 2 Laboratory of Chemical Physics, NIDDK, National Institutes of Health, Building 5, Room 137A, Bethesda, MD 20892-0520, USA; 3 MRC Centre for Protein Engineering, Hills Rd, Cambridge CB2 2QH, UK

**Keywords:** fnIII, fibronectin-type III, FNfn10, 10th fnIII domain of human fibronectin, TNfn3, third fnIII domain of human tenascin, FNoTNc, a core-swapped protein with the “outside” (surface and loops) of FNfn10 and the core of TNfn3, GdmCl, guanidinium chloride, HSQC, heteronuclear single quantum coherence, TOCSY, total correlated spectroscopy, protein folding, side-chain dynamics, immunoglobulin, extracellular matrix, protein design

## Abstract

Two homologous fibronectin type III (fnIII) domains, FNfn10 (the 10th fnIII domain of human fibronectin) and TNfn3 (the third fnIII domain of human tenascin), have essentially the same backbone structure, although they share only ∼ 24% sequence identity. While they share a similar folding mechanism with a common core of key residues in the folding transition state, they differ in many other physical properties. We use a chimeric protein, FNoTNc, to investigate the molecular basis for these differences. FNoTNc is a core-swapped protein, containing the “outside” (surface and loops) of FNfn10 and the hydrophobic core of TNfn3. Remarkably, FNoTNc retains the structure of the parent proteins despite the extent of redesign, allowing us to gain insight into which components of each parent protein are responsible for different aspects of its behaviour. Naively, one would expect properties that appear to depend principally on the core to be similar to TNfn3, for example, the response to mutations, folding kinetics and side-chain dynamics, while properties apparently determined by differences in the surface and loops, such as backbone dynamics, would be more like FNfn10. While this is broadly true, it is clear that there are also unexpected crosstalk effects between the core and the surface. For example, the anomalous response of FNfn10 to mutation is not solely a property of the core as we had previously suggested.

## Introduction

Studies of the folding of structurally related proteins have been a powerful tool for investigating conservation of folding pathways,^1^ identifying structurally important residues,^2^ and examining the role of highly conserved residues.^3^ One of the most common folds in the SCOP database^4^ is the immunoglobulin (Ig)-like fold. Over 40,000 Ig-like domains have been identified in the current PFam database^5^ and the fold is found in a number of different superfamilies, where there is no apparent sequence identity between superfamilies.^4^ In this study, rather than seeking to understand what related proteins have in common, we ask a different question—can differences between closely related proteins be explained?

Many members of the Ig-like fold have been well characterised, leading to: identification of the key residues essential for formation of the Greek key structure,^6,7^ the observation of a correlation between stability and folding rate,^8–11^ the identification of a common folding pathway,^12–16^ and the identification of the role of conserved proline residues^3^ and the conserved tyrosine corner motif.^2^ However, when two Ig-like domains from the fibronectin type III (fnIII) superfamily, the 10th fnIII domain of human fibronectin (FNfn10) and the third fnIII domain of human tenascin (TNfn3), were studied in detail and compared, they were found to differ markedly in several respects. FNfn10 and TNfn3 are essentially structurally identical (backbone RMSD is 1.2 Å), but have low sequence identity (24%). They differ in their stabilities^17,18^ and response to mutation^19^; they differ in their folding kinetics^17,18,20^ (although they share a common folding mechanism^12,16^); they display different backbone and side-chain dynamics^21–23`^; and, finally, they differ in their response to mechanical force.^24–26^ Why do proteins that are structurally almost identical behave so differently? Which components of a protein are responsible for the various aspects of its behaviour? How independent are the properties of the surface and hydrophobic core? We have addressed these questions by making a chimera of these two fnIII domains.

**Fig. 1:**
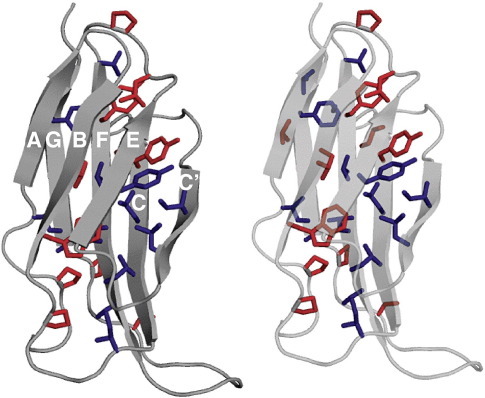
Stereo view of the structure of FNoTNc showing all the core residues. The residues that are the same in FNfn10 and TNfn3 are coloured red; the residues that were substituted are shown in blue.

The chimera, FNoTNc, was created with the “outside” (surface and loops) of FNfn10 and the core of TNfn3.^26^ Fifteen mutations were made in the core of FNfn10 so that all buried residues (with < 10% solvent-accessible surface area) are identical with those residues in the core of TNfn3 (total number of core residues = 27) (Fig. 1). Thus, we can test how transferable the properties of the core and surface of the respective parents are when combined in this way. FNoTNc is a stable, folded protein that is structurally almost identical with the parent proteins (Supplementary Fig. 1). FNoTNc has also retained the cell-adhesion activity of the FNfn10 parent protein mediated by specific integrin binding.^26^ We have previously used this chimera to demonstrate that resistance to mechanical force is a core property: FNoTNc has mechanical unfolding properties indistinguishable from TNfn3 and very different from FNfn10.^26^ Here we investigate the stability, folding and dynamics of the new, chimeric protein.

Most protein engineering analyses concentrate on the hydrophobic core of proteins, since it was shown in 1959 that the hydrophobic interaction is the major factor involved in protein folding^27^ and surface mutations rarely affect protein stability by more than ∼ 1 kcal mol^− 1^. Our results suggest, however, that the surface and loops can play a key (and sometimes unexpected) role in determining the biophysical properties of a protein.

## Results

### Thermodynamic stability

#### Wild-type FNoTNc

FNoTNc has a free energy of unfolding (Δ*G*_D–N_) of 7.5 kcal mol^− 1^, intermediate between the stabilities of FNfn10 and TNfn3 (9.4 and 6.7 kcal mol^− 1^, respectively) at pH 5.0. We infer that the surface interactions and loop and turn regions of FNfn10 make a significant contribution to the overall stability of FNoTNc. As was shown for FNfn10, the Δ*G*_D–N_ of FNoTNc is independent of pH between pH 5.0 and pH 7.0, whereas TNfn3 is less stable at pH 7 (5.7 kcal mol^− 1^). The dependence of the stability of TNfn3 on pH has been shown to be the result of the presence of patches of acidic residues on the surface of TNfn3, which are, of course, not present in FNoTNc.^17^

#### Anomalous response of certain peripheral mutations

**Fig. 2:**
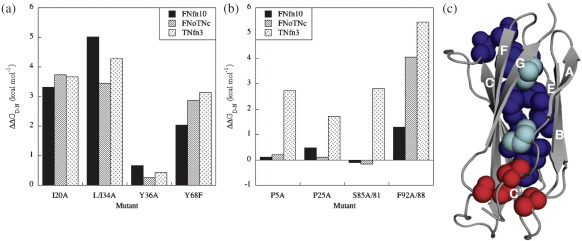
Response of FNoTNc to mutation. Histograms of (a) central core mutations and (b) peripheral *versus* FNfn10 and TNfn3. Note that mutation of residues Pro5, Pro25 and Ser85 in FNoTNc causes little loss of stability (as in FNfn10), whereas mutation of Phe92 (and Ile8, data not shown) results in a loss in stability close to that seen in TNfn3. (c) Backbone ribbon representation of FNoTNc created using MacPyMOL. Peripheral core residues P5, P25 and S85, which show little loss in stability in FNoTNc are coloured red, residues I8 and F92 are coloured cyan and other core residues are coloured blue. Data for FNfn10 and TNfn3 are taken from Ref. 19. Note that the ΔΔ*G*_D–N_ for I20A in FNfn10 was incorrectly reported in the original work.^19^ This has been remeasured for this study.

A number of core residues were mutated in FNoTNc to investigate the response of the protein to mutation. These were positions that had previously been investigated in the parent proteins FNfn10 and TNfn3. The thermodynamic stability of the mutant proteins was determined by chemical denaturation in guanidinium chloride (GdmCl). The mutations can clearly be divided into two categories. A few peripheral mutations (mutations in the A and G strands and the B–C loop) have little effect on stability as was previously observed in FNfn10 (Fig. 2a), whereas most other mutations were more typically destabilising. In the latter case, the ΔΔ*G*_D–N_ was similar to (but in general slightly lower than) what had been observed previously in TNfn3 (Fig. 2b). The residues in each category are mapped onto a backbone ribbon representation of FNoTNc in Fig. 2c. ΔΔ*G*_D–N_ values for all mutations are compared to those of FNfn10 and TNfn3 in Supplementary Table 1.

#### Equilibrium hydrogen exchange

**Fig. 3:**
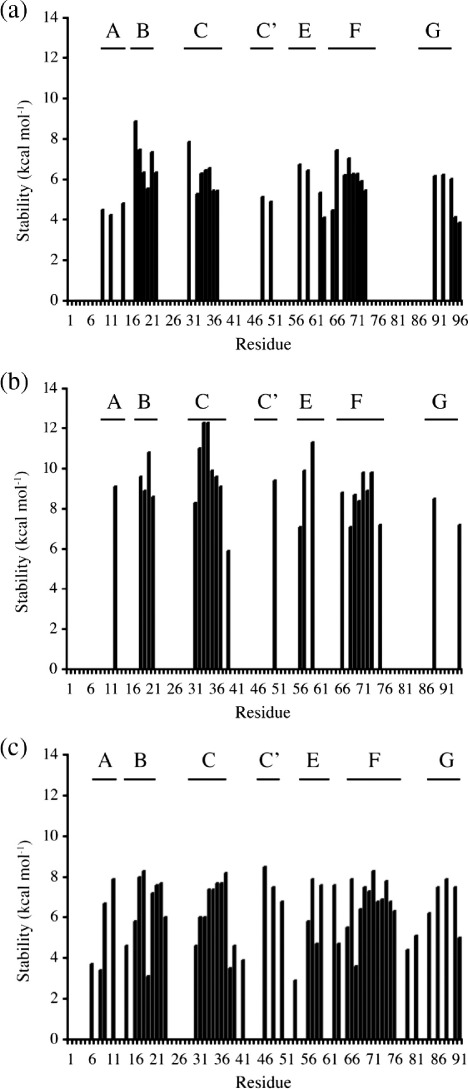
Free energy of exchange for backbone amides. Hydrogen exchange measurements were all made at pH 7.0, in 50 mM phosphate buffer and at 25°C (EX2 conditions). (a) FNoTNc, (b) FNfn10 and (c) TNfn3. The β-strands are indicated above the graphs. The values for residues L19 in FNoTNc and R33 in FNfn10 have a larger error associated with them as the exchange was not complete at the time of the experiment. Data for FNfn10 and TNfn3 were taken from Ref. 19.

The rates of hydrogen–deuterium exchange were measured at pD 7.0. Measured rate constants for exchange, *k*_ex_, ranged between 9.3 × 10^− 2^ and 1.6 × 10^− 4^ min^− 1^. Values of *k*_ex_ and the apparent free energies of exchange, Δ*G*_ex_^app^, are listed in Supplementary Table 2. The Δ*G*_ex_^app^ of FNoTNc, FNfn10 and TNfn3 are compared in Fig. 3. The hydrogen exchange behaviour of FNoTNc clearly resembles that of FNfn10: both have many residues that exchange in the experimental dead time, in particular in the edge strands (A, C′, E and G strands) that are more protected in TNfn3. Note that this does not result from differences in the intrinsic stabilities of the domains—TNfn3 is significantly less stable than either FNoTNc or FNfn10 under these experimental conditions.

### Folding kinetics

#### Wild-type FNoTNc

**Fig. 4:**
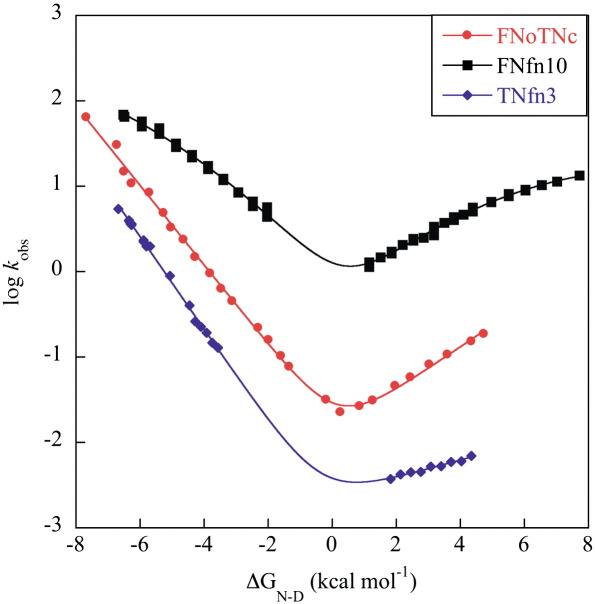
Folding kinetics of FNoTNc, FNfn10 and TNfn3. The logarithm of the rate constant (s^−^ ^1^) is plotted against the stability. FNoTNc measurements are made in GdmCl, FNfn10 in guanidine isothiocyanate and TNfn3 in urea. Data for FNfn10 and TNfn3 were taken from Refs. 17,18.

The rate constants of folding and unfolding were determined using stopped-flow measurements monitored by changes in fluorescence > 320 nm. The logarithm of the folding and unfolding rate constants was plotted against concentration of denaturant (Fig. 4). Both unfolding and refolding arms show a linear dependence on denaturant concentration. There is an additional refolding phase, which we attribute to proline isomerisation and do not consider further. (Both FNfn10 and TNfn3 have proline-isomerisation limited phases, and FNoTNc has eight Pro residues.) To compare the kinetics of FNoTNc with those of FNfn10 and TNfn3 (which have been studied using different denaturants due to large differences in stability), the logarithm of the observed rate constants is plotted against stability in Fig. 4. The free energy of unfolding calculated from the ratio of the folding and unfolding rate constants extrapolated to 0 M denaturant (7.7  kcal mol^− 1^) and the kinetic *m* value (2.1 kcal mol^− 1^ M^− 1^) are the same as the equilibrium Δ*G*_D–N_ (7.5 kcal mol^− 1^) and *m* value (2.1 kcal mol^− 1^ M^− 1^) within error—consistent with the folding being a 2-state process, with no stable intermediates being populated. The relative compactness of the transition state can be determined from the folding and unfolding *m* values. FNoTNc has a β_T_ value of 0.6, similar to those of TNfn3 and FNfn10.

#### Φ-value analysis

The folding kinetics of 18 variants of FNoTNc with nondisruptive deletion mutations were measured as for the wild type. Few of these mutants were in the A, B and G strands due to very small changes in stability with mutation in general. In a few of the mutants, the unfolding arm of the chevron plots shows negative curvature at high denaturant concentrations, which we attribute to the Hammond effect: the simplest model that fits the data.^28–30^ All chevrons were fitted therefore to a two-state equation with a quadratic term in the unfolding arm. (All the chevron plots are shown in Supplementary Fig. 2.) Note that the model chosen to fit the kinetic data does not affect our results because equilibrium values of ΔΔ*G*_D–N_ were used to determine Φ. In Table 1, the Φ values for each mutant are compared to those in TNfn3 and FNfn10. The Φ values are generally low, closely resembling those of TNfn3.

### Dynamics

#### Backbone dynamics

**Fig. 5:**
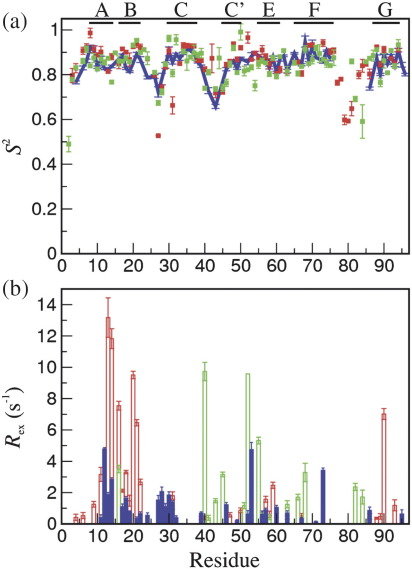
Backbone dynamics. Comparison of model-free parameters of TNfn3 (green), FNfn10 (red) and FNoTNc (blue). (a) Generalised order parameters, *S*^2^. (b) Conformational exchange terms, *R*_ex_. Gaps indicate missing data. Data for FNfn10 and TNfn3 were taken from Ref. 22.

^15^N *T*_1_ and *T*_2_ relaxation time constants and ^1^H^15^N nuclear Overhauser enhancement parameters were measured using ^1^H–^15^N correlation spectroscopy (Supplementary Table 3). The generalised order parameter, *S*^2^, and a conformational exchange broadening parameter, *R*_ex_, were determined for each backbone amide (Fig. 5 and Supplementary Table 4). The *S*^2^ values are similar to those of both the parents. However, the high values of *R*_ex_ that were seen in the A/B region of FNfn10, have decreased in FNoTNc.

#### Side-chain dynamics

**Fig. 6:**
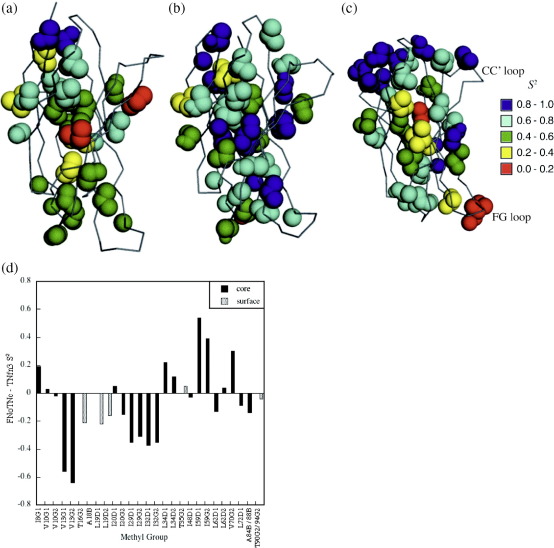
Side-chain dynamics. The methyl *S*^2^ values of (a) FNoTNc (b) FNfn10 and (c) TNfn3 are shown projected onto the protein structure. The three structures are in approximately the same orientation with the C-terminus at the top of the molecule. *S*^2^ ranges from zero to unity, with higher values of *S*^2^ indicating greater conformational restriction. FNoTNc has a cluster of highly mobile residues in the core as does TNfn3. Figures created using MacPyMOL. (d) A comparison of *S*^2^ values of identical side chains in FNoTNc and TNfn3. (A similar comparison has not been shown for FNfn10 and FNoTNc since very few core residues have the same identity and so the side-chain dynamics per residue are not directly comparable.) Data for FNfn10 and TNfn3 were taken from Ref. 22.

The relaxation of the operator terms *I*_*z*_*C*_*z*_*D*_*z*_, *I*_*z*_*C*_*z*_*D*_*y*_ and *I*_*z*_*C*_*z*_ was measured to give deuterium relaxation time constants *T*_1_(D) and *T*_2_(D)^31^ (Supplementary Table 5). Order parameters, *S*^2^, were determined for each methyl group using the standard model-free formalism as previously described^22^ (Supplementary Table 6). The dynamics data for those methyl groups that had overlapping peaks were treated with caution, as the contribution from the overlapped peaks cannot be separated. Nevertheless, we have some confidence in these results due to the agreement of the order parameters for residues that have both overlapped and well-resolved methyl groups. *S*^2^ ranges from zero to unity, with higher values of *S*^2^ indicating greater conformational restriction. The methyl *S*^2^ values are shown projected onto the protein structure and compared to the parent proteins in Fig. 6. Within the core of FNoTNc is a cluster of deeply buried residues, which have unusually low order parameters, as has previously been observed in TNfn3.

## Discussion

The core of FNoTNc is similar to that of TNfn3, but apparently less closely packed.

### Evidence from mutations

**Fig. 7:**
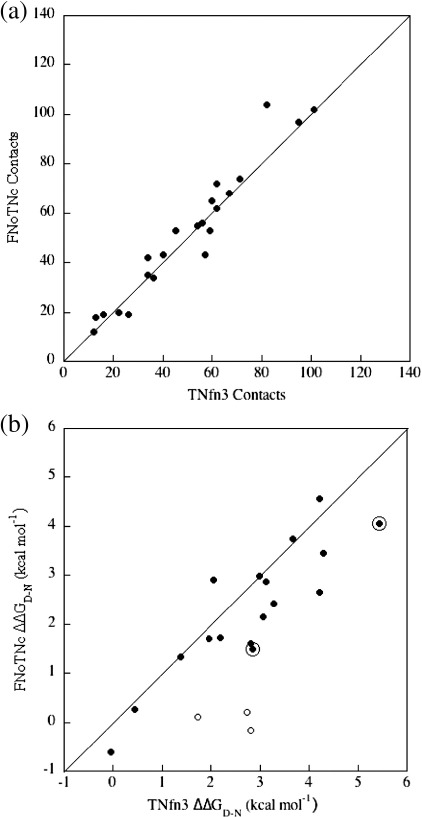
Comparison of the cores of FNoTNc and TNfn3. (a) Core packing, number of side chain–side chain contacts (within 6 Å) made by each residue (b) Comparison of ΔΔ*G*_D–N_ values. Peripheral core residues P5, P25 and S85, which show little loss in stability in FNoTNc (as in FNfn10) are shown as open circles, while all other residues are shown as filled circles. Residues I8 and F92 which have a change in stability close to that seen in TNfn3 and greater than that in FNfn10 are ringed (see the text). Stability data for FNfn10 and TNfn3 were taken from Ref. 19.

FNoTNc is close in structure to both the parent domains (backbone RMSD, excluding the mobile C–C′ and F–G loops, is 0.95 and 0.89 Å compared to FNfn10 and TNfn3, respectively). From the crystal structure we would deduce that the core of FNoTNc is essentially the same in terms of core packing and number of core contacts as TNfn3, the parent protein that donated the core residues (Fig. 7a). However, the calculated free volume in the interior of FNoTNc is larger than for TNfn3 (128.4 Å^3^ *versus* 118.1 Å^3^, respectively). When the response of FNoTNc to mutation is compared to TNfn3, it is clear that the ΔΔ*G*_D–N_ values of FNoTNc are slightly lower, on average ∼ 80% of those in TNfn3 (Fig. 7b). Since the loss of free energy on mutation is strongly correlated with the number of side-chain contacts deleted,^32,33^ we infer that the less tight packing of the core of FNoTNc accounts for the difference in ΔΔ*G*_D–N_ compared to TNfn3.

### Evidence from side-chain dynamics

The same conclusion can be drawn from the analysis of the side-chain dynamics. The core of TNfn3 has been shown to be exceptionally mobile, with several of the most buried residues falling several standard deviations below the expected order parameter for their residue type.^22,23^ FNfn10 has a core that is much more conformationally restricted, with order parameters within the usual range for buried residues. Our previous analysis of the side-chain dynamics of FNfn10 and TNfn3 led us to suggest that the differences in core dynamics between FNfn10 and TNfn3 could, at least in part, be ascribed to the slightly lower core packing in TNfn3; the residues in TNfn3 with unusually low order parameters are also found to have packing volumes that are larger than expected. The buried side chains of FNoTNc were found to have, on average, even lower order parameters than the same side chains in TNfn3 (Fig. 6d).

### Behaviour of peripheral regions of the protein is modulated by the surface and loops

#### Evidence from mutation

A number of sites in FNfn10 were identified where upon mutation the ΔΔ*G*_D–N_ is significantly lower than for the same (or an equivalent) mutation in TNfn3.^19^ These sites are at the periphery of the protein and include residues Pro5 and Leu8 (A strand), Pro25 (B–C loop) and residues Ser85 and Phe92 (G strand). Unexpectedly, in FNoTNc we see the same anomalous response to mutation for Pro5, Pro25 and Ser85, although Ile8A and Phe92A now have similar ΔΔ*G*_D–N_ values to TNfn3 (Fig. 2a). Pro5, Pro25 and Ser85 are all found in the same region of the molecule (Fig. 2c). The anomalous response to mutation in this peripheral region of the core of FNoTNc and FNfn10 is intriguing: apparently the “plastic” response of the protein to mutation of these three buried residues is modulated by the surface of the protein and is not determined by the core alone. The additional plasticity at this end of the hydrophobic core may be due to its proximity to the longer FG loop in FNfn10 and FNoTNc, which restricts motion less than the corresponding loop in TNfn3.

#### Evidence from hydrogen exchange

FNoTNc, FNfn10 and TNfn3 have very similar structures and hydrogen bonding patterns. Further evidence for “plasticity” of FNfn10 compared to TNfn3 came from the observation that the peripheral A, C′, E and G strands were significantly less well protected against amide exchange in FNfn10 than in TNfn3, despite the fact that FNfn10 is considerably more stable than TNfn3.^19^ Under the experimental conditions, hydrogen exchange is in the EX2 regime,^34,35^ meaning that exchange reflects local stability; that is, these peripheral regions of FNfn10 have lower local stabilities than TNfn3. Our experiments clearly show that the exchange behaviour of FNoTNc is similar to FNfn10—with low protection of residues in the same peripheral strands (Fig. 3). Again surprisingly, local instability appears to be a function of the surface of the protein and not a property of the core; however, the plasticity inferred from response to mutation and local instability still appear to be related, as was previously inferred. This is not, however, related to slow exchange motions of the backbone, as we had previously suggested, since the millisecond time scale motions observed in the A/B region of FNfn10 are not found in FNoTNc.

### The stability of FNoTNc is modulated by both the core and the surface

In summary, despite the evidence for FNoTNc having a less well packed core than TNfn3, with the mutation of buried side chains having less effect on the overall stability (Fig. 7b), FNoTNc is significantly more stable than TNfn3, suggesting again that the surface and loops of FNfn10 contribute significantly to the stability of FNoTNc. This is not a new observation; it has been previously demonstrated that surface interactions can play a significant role in the stabilisation of proteins.^36^ Interestingly, however, the “local stability” of the peripheral regions of FNoTNc is lower than the local stability of TNfn3, as manifested in the hydrogen exchange experiments. The surface residues of FNfn10 are modulating the stability of these peripheral regions.

A concurrent study by Siggers *et al.*^37^ investigated the stability and dynamics of a series of loop-swap mutants of FNfn10 and TNfn3: essentially these were mutants where the C–C′ and F–G loops were exchanged. Interestingly, both TNfn3 domains had lower thermal stabilities than the wild-type protein, whereas the thermal stability of FNfn10 was unaffected by the exchange. We infer that in our chimeric protein, interactions of the surface residues are most important in the increase in stabilisation of FNoTNc beyond that of TNfn3, and not the new C–C′ and F–G loops.

### The TNfn3 core governs the folding kinetics

#### Wild-type kinetics

In order to compare the kinetics of the three proteins directly, the logarithm of the rate constant has been plotted against stability (Fig. 4). This unusual scale is used as each protein was characterised using a different denaturant. FNoTNc folds at a rate intermediate between FNfn10 and TNfn3. In broad terms, the chevron of FNoTNc resembles that of TNfn3. It has previously been pointed out that the folding rates of Ig-like domains in water do not correlate with contact order (the average sequence separation of native contacts).^8,38^ For the present three proteins, which have almost identical contact order (absolute contact order 15–16%, relative contact order 16–17%), the rate constants at the folding midpoint span 2.5 orders of magnitude. Thus, the variation in the folding rate cannot be explained only by differences in contact order—protein stability plays a key role.^8,39^

FNfn10 shows clear rollover in both the folding and unfolding arms. The rollover in the folding arm has previously been ascribed to the presence of a populated folding intermediate.^18^ Neither FNoTNc nor TNfn3 show any evidence for population of a folding intermediate—the presence of a stable folding intermediate in FNfn10 appears to be the result of core interactions.

Curvature in the unfolding arm of a chevron plot has been ascribed to the presence of a high-energy intermediate, to Hammond effects, or to population of an unfolding intermediate.^30,40,41^ This has not been investigated for any of these fnIII domains; indeed, it is often not possible to distinguish between the first two cases.^42^ Both FNfn10 and some mutants of FNoTNc display curvature in the unfolding arm that is not seen in wild-type TNfn3. It should be noted, however, that unfolding curvature has been seen in a less stable form of TNfn3 (missing the final two C-terminal residues) and may simply not be observed in this case because the unfolding arm in the stable form of TNfn3 used here is relatively short. In summary, FNoTNc has similar folding characteristics to TNfn3. This suggests that the core of these proteins plays the major role in determining the folding behaviour.

However, FNoTNc is more stable than TNfn3. When we compare the folding and unfolding rate constants in water we find that FNoTNc folds some 10 times faster than TNfn3 (*k*_f_^H_2_O^ = 60 and 6 s^− 1^, respectively) but unfolds at approximately the same rate as TNfn3 (*k*_u_^H_2_O^ = 2 × 10^− 4^ and 5 × 10^− 4^ s^− 1^, respectively). Thus, the stabilising surface interactions apparently stabilise the transition state of FNoTNc as much as the native state, while still not causing a folding intermediate to be populated. This, perhaps, allows us to pinpoint further which surface interactions are responsible for the added stability of FNoTNc (over TNfn3). At the transition state, the loops and the peripheral A and G strands are largely unstructured, but there is structure in the B, C, C′, E and F strands, particularly towards the centre of the core (see Φ value section below). Interactions between surface residues in these strands are therefore the most likely candidates for providing additional stability to the FNoTNc protein (both native and transition states), above those interactions between residues that are packing in the core.

In contrast, FNoTNc folds some three times more slowly than FNfn10 and the unfolding rate constant of FNfn10 in water is approximately an order of magnitude lower (∼ 2 × 10^− 5^ s^− 1^).

#### Φ-value analysis

The Φ-value analysis reveals the extent of formation of structure in the transition state.^43^ The pattern of Φ values in FNfn10 and TNfn3 are similar, suggesting that they have a common folding mechanism. There are differences, however. The Φ values of TNfn3 tend to be much lower than those of FNfn10: TNfn3 has five Φ values greater than 0.4,^12^ while FNfn10 has eight^16^; in FNfn10 the folding nucleus appears to be more extensive than in TNfn3, with more than one residue in the central C, E and F strands having high Φ values. FNoTNc has even lower average Φ values than TNfn3 with only two Φ values greater than 0.4 (Table 1) Both FNoTNc and TNfn3 have less extensive formation of structure in the transition state than FNfn10.

**Fig. 8:**
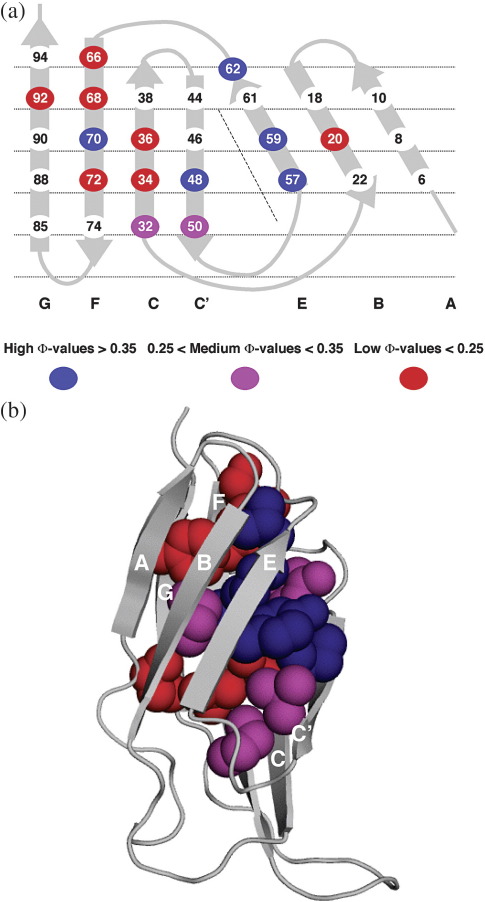
FNoTNc Φ values. (a) Φ value cartoon. The ribbon structure is shown “opened out” with the core packing layers defined by the horizontal lines. (b) The Φ value for each residue is shown mapped onto a backbone ribbon representation of the structure. Figure created in MacPyMOL. Low Φ values (< 0.25) are coloured red, medium Φ values (0.25 < Φ > 0.35) are coloured magenta and high Φ values (> 0.35) are coloured blue.

However, Φ-value analysis is at its most powerful when patterns of Φ values are compared in different proteins rather than by direct comparison of Φ values. Previous analysis of TNfn3 has identified a ring of interacting residues in the B, C, E and F strands as the folding nucleus, with residues in the C′ strand packing onto these.^12^ The Φ values of the peripheral A and G strands are all close to 0, suggesting that they are unformed at the transition state. A similar pattern of Φ values was seen in FNfn10, although this Φ-value analysis was less complete.^16^ The Φ values for FNoTNc were divided into three categories of low (Φ < 0.25), medium (0.25 < Φ < 0.35) and high (Φ > 0.35) (Fig. 8) and compared to the pattern of Φ values for the identical residues in TNfn3. The pattern of Φ values is very similar to that of TNfn3, although there are slight qualitative differences. Again, the highest Φ values are found in the B, C, C′, E and Φ strands and the Φ values in the A and G strands are ∼ 0. The folding mechanism of FNoTNc is unperturbed and the same as both the parents, although from the magnitude and extent of the residues with higher Φ values the transition-state structure appears to be closer to that of TNfn3 than FNfn10.

### Dynamics from NMR is determined by local interactions

#### Side-chain dynamics

The side-chain dynamics appear to reflect core packing, as was discussed above, and so FNoTNc resembles TNfn3 more closely than FNfn10.

#### Backbone dynamics

The *S*^2^ values of FNoTNc are generally very similar to those of both the parents (Fig. 5). It is interesting to compare our study with that of the loop swap mutants of FNfn10 and TNfn3 from Palmer and coworkers,^37^ where the F–G loop of FNfn10 was grafted into TNfn3. In that case, the *S*^2^ values in the loop region were more similar to those in FNfn10, than those in the equivalent positions in TNfn3. A similar result was seen in the protein where the C–C′ loop was grafted in. It is difficult to compare our results directly to those of Siggers *et al.*^37^: their method of analysis leads to greater differences between the *S*^2^ values of FNfn10 and TNfn3, resulting in more visible effects on the *S*^2^ values when the loops are swapped than we see in FNoTNc compared to the parent proteins. However, there is a general agreement that the behaviour of the loops is a local property, rather than being a direct effect of either core or surface interactions.

## Conclusion

We have grafted the core of one fnIII domain (TNfn3) into the homologue FNfn10, creating a chimera, FNoTNc, which has retained the structure of the parent proteins. Using several different probes, we have shown that FNoTNc does not behave like either one of the parent proteins alone. Instead, it has retained a number of properties of each. We find that each property investigated clearly resembles the behaviour of one of the parents, enabling us to separate the contribution of the core and the surface of the protein in determining the behaviour of the domain. Some of these are unsurprising, such as the pH dependence of stability, the core side-chain dynamics and the dependence of folding on the composition of the core. However, the surface of the protein confers significant stability not only on the native state, but also on the transition state for folding. Others properties are less predictable. The surface of the protein confers “plasticity” in peripheral regions of the proteins as detected by the anomalous response of some regions of the core to mutation and hydrogen exchange protection patterns.

This suggests that the surface of a domain may have a more significant coupling with the core than we had previously considered. Since most biophysical studies tend to focus on the core of a protein, this coupling is a relatively unexplored area of research.

## Materials and Methods

### Chemicals

GdmCl was purchased from MP Biomedicals Inc., guanidine isothiocyanate from Gibco-BRL and urea from BDH Laboratory Supplies.

### Protein expression and purification

Site-directed mutagenesis reactions were performed with the QuickChange kit from Stratagene using the FNoTNc plasmid. The identity of the mutants was confirmed by DNA sequencing. The mutants studied in this work are listed in Table 1 and Supplementary Table 1. The nature of the mutation is indicated with the wild-type residue first (single-letter code), the position of the mutation second and the mutant residue third. Expression and purification of FNoTNc and mutants was performed as described earlier for TNfn3.^20^

### Measurements of protein stability

All biophysical measurements were performed in 50 mM sodium acetate buffer, pH 5.0 at 25 °C unless otherwise stated. The stability of FNoTNc and FNoTNc mutants were determined by equilibrium denaturation experiments using GdmCl and by standard methods using 1 μM protein.^20^

### Kinetic measurements

Kinetics were measured using fluorescence stopped-flow measurements in 50 mM sodium acetate buffer, pH 5.0, at 25 °C and were monitored by changes in fluorescence above 320 nm. Refolding measurements for FNoTNc were made in 0 M denaturant by stopped-flow fluorescence using pH jumps from pH 12.4 to pH 5.0 as previously described.^12^ (FNoTNc is acid stable.) NaOH (25 mM, pH 12.4) unfolds FNoTNc completely (data not shown).

### Φ-value analysis

Φ values were determined from refolding data at 1.0 M GdmCl, to avoid the errors associated with long extrapolation, from Eq. (1):^43^

(1) $$\Phi =\frac{\Delta \Delta G_{\text{D}–\text{TS}}}{\Delta \Delta G_{\text{D}–\text{N}}}$$

where

$$\Delta \Delta G_{\text{D}–\text{TS}}=RT\ln\left(\frac{k_{\text{f}}^{\text{WT}}}{k_{\text{f}}^{\text{mut}}}\right)$$

and *k*_f_^WT^ and *k*_f_^mut^ are the rate constants for folding of wild-type and mutant proteins, respectively.

### NMR sample preparation

FNoTNc was expressed and purified by affinity chromatography as previously described.^17^ Uniformly ^15^N labelled and ^13^C and ^15^N labelled samples were expressed in M9 minimal media containing ^15^NH_4_Cl and [U-^13^C]6-glucose as the sole nitrogen and carbon sources. Samples for side-chain dynamics were expressed as previously described.^22^

### Chemical shift assignments

Backbone assignment experiments were carried out on a double-labelled (^13^C, ^15^N) sample of FNoTNc, at an approximate concentration of 1–2 mM, in 50 mM imidazole buffer at pH 7.0 in 10% D_2_O. Sodium azide (0.05%) was added to prevent microbial growth. The sample was centrifuged to remove insoluble protein and degassed. Spectra were acquired at 298 K on a Bruker DRX500 spectrometer with an inverse triple-resonance cryogenic probe. Backbone assignments were based on HNCACB, HNCO and CBCA(CO)NH experiments together with the ^1^H–^15^N heteronuclear single quantum coherence (HSQC) spectrum. Side-chain ^1^H and ^13^C resonance assignments were obtained from 3-D HCCH–total correlated spectroscopy (TOCSY) H(CCCO)NH and (H)CC(CO)NH preceding TOCSY spectra. The spectra were processed and analysed using NMRpipe and Sparky.^44,45^ The ^1^H–^15^N HSQC has excellent resolution, although resonances from 12 residues in loop regions cannot be detected at either pH 5 or pH 7 (Supplementary Fig. 3 and Supplementary Table 7).

All samples for side-chain methyl assignment were prepared in 50 mM sodium acetate buffer at pH 5.0 in 10% D_2_O, at a concentration of 1–2 mM. Sodium azide was added to prevent microbial growth. The sample was centrifuged to remove insoluble protein and degassed. All experiments were carried out as previously described.^22,46,47^

Chemical shift assignments of the side chains were made using standard triple-resonance experiments. Many of the signals in the ^1^H–^13^C HSQC are overlapped, meaning that 5 of the 64 methyl groups in the ^1^H–^13^C HSQC could not be assigned with confidence. Assignment of the leucine and valine methyl groups was made stereospecifically, based upon the phase of peaks in a ^1^H–^13^C HSQC acquired for a sample with 10% ^13^C enrichment (Supplementary Fig. 4 and Supplementary Table 7).

### Hydrogen exchange

Hydrogen exchange experiments were carried out under EX2 conditions on a ^15^N-labelled sample of FNoTNc, at an approximate concentration of 1–2 mM, in 50 mM imidazole buffer at pD 7.0 in 10% D_2_O. Sodium azide (0.05%) was added to prevent microbial growth. The exchange of amide protons was followed by the decay of intensity of peaks in HSQC spectra.^48^

The apparent free energy of exchange, Δ*G*_ex_^app^, was determined from the rate constant of exchange, *k*_ex_, and the intrinsic rate constant, *k*_int_, determined from peptide data to take account of the primary sequence of the protein and exchange conditions^49,50^ using Eq. (2) and intrinsic rate constants determined using the software Sphere†.^51^

(2) $$\Delta G_{\text{ex}}^{\text{app}}=-RT\ln\frac{k_{\text{ex}}}{k_{\text{int}}}$$

### Backbone ^15^N relaxation measurements

Backbone dynamics were determined from ^15^N *T*_1_ and *T*_2_ relaxation times and the steady-state heteronuclear ^1^H^15^N nuclear Overhauser enhancement at 500 MHz as previously described.^22^ The data were analysed using standard protocols for backbone dynamics with the program TENSOR2.

### Side-chain methyl ^2^H relaxation measurements

Side-chain deuterium relaxation times *T*_1_(D) and *T*_1ρ_(D) were determined by measuring the relaxation of the two- and three-spin operator terms, *I*_z_*C*_z_, *I*_z_*C*_z_*D*_z_ and *I*_z_*C*_z_*D*_y_ and analysed as previously described.^22,31^

## Figures and Tables

**Table 1:** Φ values in FNoTNc, FNfn10 and TNfn3

Mutation	Position (β-strand or loop)	FNoTNc *k*_f_ (at 1 M GdmCl) (s^− 1^)	Φ values		
			FNoTNc	FNfn10^a^	TNfn3
WT		6.3 ± 0.3			
I8A	A	4.8 ± 0.5	0.1		0.1
L8A	A			ND	
I20A	B	1.0 ± 0.1	0.2	ND	0.4
I20V	B	4.7 ± 0.2	0.3	0.2	ND
I32A	C	1.5 ± 0.1	0.3		0.2
Y32A	C			0.5	
L34A	C	1.5 ± 0.1	0.2		0.4
I34A	C			0.7	
Y36A	C	2.5 ± 0.1	0.2	0.4	0.5
Y36F	C	9.0 ± 0.6	ND	ND	ND
F36A	C	2.5 ± 0.1	0.3	0.5	0.6
I48A	C′	1.4 ± 0.1	0.5		0.7
F48A	C′			0.4	
L50A	C′	1.3 ± 0.1	0.3		0.4
V50A	C′			0.6	
Y57A	E	2.7 ± 0.1	0.4		0.4
A57G	E			0.3	
I59A	E	1.0 ± 0.1	0.4	0.3	0.6
L62A	E	0.4 ± 0.1	0.4	0.3	0.3
T66A	E	5.0 ± 0.2	0.1		0.3
V66A	E			ND	
Y68F	F	3.0 ± 0.1	0.2	0.4	0.4
V70A	F	1.5 ± 0.1	0.5		0.5
I70A	F			0.6	
L72A	F	2.6 ± 0.1	0.2		0.3
V72A	F			0.6	
F92A/F88A^b^	G	2.4 ± 0.1	0.1	ND	0.1

Φ values were determined from refolding kinetics at 1.0 M GdmCl to avoid extrapolation to 0 M denaturant, which might introduce error. ND: the ΔΔ*G*_D–N_ was too low to determine a Φ value accurately.

a FNfn10 and TNfn3 data were taken from Refs. 12,16. The error in Φ is < 0.1 in all cases.

b Numbering of proteins is the same between positions 1 and 78. There is a four-residue insertion in the F–G loop of FNoTNc and FNfn10, resulting in the numbering of equivalent residues in the G strand being four numbers lower in TNfn3.

## Footnotes

† http://www.fccc.edu/research/labs/roder/sphere

## References

[1] Zarrine-Afsar A., Larson S.M., Davidson A.R. (2005). The family feud: do proteins with similar structures
fold *via* the same pathway?. Curr. Opin. Struct. Biol..

[2] Hamill S.J., Cota E., Chothia C., Clarke J. (2000). Conservation of folding and stability within a protein
family: the tyrosine corner as an evolutionary
cul-de-sac. J. Mol. Biol..

[3] Steward A., Adhya S., Clarke J. (2002). Sequence conservation in Ig-like domains: the role of
highly conserved proline residues in the fibronectin type III
superfamily. J. Mol. Biol..

[4] Murzin A.G., Brenner S.E., Hubbard T., Chothia C. (1995). SCOP: a structural classification of proteins database
for the investigation of sequences and structures. J. Mol. Biol..

[5] Bateman A., Coin L., Durbin R., Finn R.D., Hollich V., Griffiths-Jones S. (2004). The Pfam protein families database. Nucleic Acids Res..

[6] Chothia C., Lesk A.M., Tramontano A., Levitt M., Smith-Gill S.J., Air G. (1989). Conformations of immunoglobulin hypervariable
regions. Nature.

[7] Harpaz Y., Chothia C. (1994). Many of the immunoglobulin superfamily domains in cell
adhesion molecules and surface receptors belong to a new
structural set which is close to that containing variable
domains. J. Mol. Biol..

[8] Clarke J., Cota E., Fowler S.B., Hamill S.J. (1999). Folding studies of immunoglobulin-like beta-sandwich
proteins suggest that they share a common folding
pathway. Struct. Fold. Des..

[9] Plaxco K.W., Spitzfaden C., Campbell I.D., Dobson C.M. (1997). A comparison of the folding kinetics and
thermodynamics of two homologous fibronectin type III
modules. J. Mol. Biol..

[10] Spitzfaden C., Grant R.P., Mardon H.J., Campbell I.D. (1997). Module–module interactions in the cell binding region
of fibronectin: stability, flexibility and
specificity. J. Mol. Biol..

[11] Pozdnyakova I., Wittung-Stafshede P. (2003). Approaching the speed limit for Greek Key beta-barrel
formation: transition-state movement tunes folding rate of
zinc-substituted azurin. Biochim. Biophys. Acta.

[12] Hamill S.J., Steward A., Clarke J. (2000). The folding of an immunoglobulin-like Greek key
protein is defined by a common-core nucleus and regions
constrained by topology. J. Mol. Biol..

[13] Parker M.J., Spencer J., Clarke A.R. (1995). An integrated kinetic analysis of intermediates and
transition states in protein folding reactions. J. Mol. Biol..

[14] Fowler S.B., Clarke J. (2001). Mapping the folding pathway of an immunoglobulin
domain: structural detail from Phi value analysis and movement
of the transition state. Structure.

[15] Lorch M., Mason J.M., Clarke A.R., Parker M.J. (1999). Effects of core mutations on the folding of a
beta-sheet protein: implications for backbone organization in
the I-state. Biochemistry.

[16] Cota E., Steward A., Fowler S.B., Clarke J. (2001). The folding nucleus of a fibronectin type III domain
is composed of core residues of the immunoglobulin-like
fold. J. Mol. Biol..

[17] Hamill S.J., Meekhof A.E., Clarke J. (1998). The effect of boundary selection on the stability and
folding of the third fibronectin type III domain from human
tenascin. Biochemistry.

[18] Cota E., Clarke J. (2000). Folding of beta-sandwich proteins: three-state
transition of a fibronectin type III module. Protein Sci..

[19] Cota E., Hamill S.J., Fowler S.B., Clarke J. (2000). Two proteins with the same structure respond very
differently to mutation: the role of plasticity in protein
stability. J. Mol. Biol..

[20] Clarke J., Hamill S.J., Johnson C.M. (1997). Folding and stability of a fibronectin type III domain
of human tenascin. J. Mol. Biol..

[21] Carr P.A., Erickson H.P., Palmer A.G. (1997). Backbone dynamics of homologous fibronectin type III
cell adhesion domains from fibronectin and
tenascin. Structure.

[22] Best R.B., Rutherford T.J., Freund S.M., Clarke J. (2004). Hydrophobic core fluidity of homologous protein
domains: relation of side-chain dynamics to core composition and
packing. Biochemistry.

[23] Best R.B., Clarke J., Karplus M. (2005). What contributions to protein side-chain dynamics are
probed by NMR experiments? A molecular dynamics simulation
analysis. J. Mol. Biol..

[24] Ng S.P., Rounsevell R.W., Steward A., Geierhaas C.D., Williams P.M., Paci E., Clarke J. (2005). Mechanical unfolding of TNfn3: the unfolding pathway
of a fnIII domain probed by protein engineering. AFM and MD
simulation. J. Mol. Biol..

[25] Oberhauser A.F., Badilla-Fernandez C., Carrion-Vazquez M., Fernandez J.M. (2002). The mechanical hierarchies of fibronectin observed
with single-molecule AFM. J. Mol. Biol..

[26] Ng S.P., Billings K.S., Ohashi T., Allen M.D., Best R.B., Randles L.G. (2007). Designing an extracellular matrix protein with
enhanced mechanical stability. Proc. Natl Acad. Sci. USA.

[27] Kauzmann W. (1959). Some factors in the interpretation of protein
denaturation. Adv. Protein Chem..

[28] Hammond G.S. (1955). A correlation of reaction rates. J. Am. Chem. Soc..

[29] Matouschek A., Fersht A.R. (1993). Application of physical organic chemistry to
engineered mutants of proteins: Hammond postulate behavior in
the transition state of protein folding. Proc. Natl Acad. Sci. USA.

[30] Oliveberg M. (2001). Characterisation of the transition states for protein
folding: towards a new level of mechanistic detail in protein
engineering analysis. Curr. Opin. Struct. Biol..

[31] Muhandiram D.R., Yamazaki T., Sykes B.D., Kay L.E. (1995). Measurement of ^2^H
*T*_1_ and
*T*_1ρ_ relaxation times
in uniformly ^13^C-labeled and
fractionally ^2^H-labeled proteins in
solution. J. Am. Chem. Soc..

[32] Main E.R., Fulton K.F., Jackson S.E. (1998). Context-dependent nature of destabilizing mutations on
the stability of FKBP12. Biochemistry.

[33] Serrano L., Kellis J., Cann P., Matouschek A., Fersht A.R. (1992). The folding of an enzyme. II. Substructure of barnase
and the contribution of different interactions to protein
stability. J. Mol. Biol..

[34] Hvidt A.A., Nielsen S.O. (1966). Hydrogen exchange in proteins. Adv. Protein Chem..

[35] Linderstrøm-Lang K. (1955). Deuterium exchange between peptides and
water. Chem Soc., Spec. Publ..

[36] Perl D., Mueller U., Heinemann U., Schmidt F.X. (2000). Two exposed amino acids confer thermostability on a
cold shock protein. Nat. Struct. Biol..

[37] Siggers K.F., Soto C., Palmer A.G.R. (2007). Conformational dynamics in loop swap mutants of
homologous fibronectin type III domains. Biophys. J..

[38] Plaxco K.W., Simons K.T., Baker D. (1998). Contact order, transition state placement and the
refolding rates of single domain proteins. J. Mol. Biol..

[39] Dinner A.R., Karplus M. (2001). The roles of stability and contact order in
determining protein folding rates. Nat. Struct. Biol..

[40] Sanchez I.E., Kiefhaber T. (2003). Evidence for sequential barriers and obligatory
intermediates in apparent two-state protein
folding. J. Mol. Biol..

[41] Otzen D.E., Oliveberg M. (2002). Conformational plasticity in folding of the split
beta–alpha–beta protein S6: evidence for burst-phase disruption
of the native state. J. Mol. Biol..

[42] Scott K.A., Clarke J. (2005). Spectrin R16: Broad energy barrier or sequential
transition states?. Protein Sci..

[43] Fersht A.R., Matouschek A., Serrano L. (1992). The folding of an enzyme. I. Theory of protein
engineering analysis of stability and pathway of protein
folding. J. Mol. Biol..

[44] Delaglio F., Grzesiek S., Vuister G.W., Zhu G., Pfeifer J., Bax A. (1995). NMRPipe: a multidimensional spectral processing system
based on UNIX pipes. J. Biomol. NMR.

[45] Goddard, T. D. & Kneller, D. G. SPARKY 3. University of California, San Francisco, CA.

[46] Sattler M., Schleucher J., Griesinger C. (1999). Heteronuclear multidimensional NMR experiments for the
structure determination of proteins in solution employing pulsed
field gradients. Prog. Nuclear Magn. Reson.
Spectrosc..

[47] Neri D., Szyperski T., Otting G., Senn H., Wuthrich K. (1989). Stereospecific nuclear magnetic resonance assignments
of the methyl groups of valine and leucine in the DNA-binding
domain of the 434 repressor by biosynthetically directed
fractional ^13^C
labeling. Biochemistry.

[48] Bax A., Ikura M., Kay L.E., Torchia D.A., Tschudin R. (1990). Comparison of different modes of two-dimensional
reverse-correlation NMR for the study of proteins. J. Magn. Reson..

[49] Bai Y., Milne J.S., Mayne L., Englander S.W. (1993). Primary structure effects on peptide group hydrogen
exchange. Proteins: Struct., Funct.,
Genet..

[50] Bai Y., Milne J.S., Mayne L., Englander S.W. (1994). Protein Stability parameters measured by hydrogen
exchange. Proteins: Struct. Funct. Genet..

[51] Zhang, Y.-Z. Protein and peptide structure and interactions studied by hydrogen exchange and NMR. PhD Thesis, University of Pennsylvania.

