# Supplementary material for: Crosstalk between the Protein Surface and Hydrophobic Core in a Core-swapped Fibronectin Type III Domain
Source: J Mol Biol. 2008 Jan 11;375(2):560–71. doi: 10.1016/j.jmb.2007.10.056 (PMC2291452; doi:10.1016/j.jmb.2007.10.056)
Supplement: Supplementary Figures [file applic1.doc]

**Table S1**. Changes in stability with mutation in FNoTNc

| Mutation^a^ | Position (β-strand or loop) | FNoTNc $\text{DD}G_{\text{D-N}}$ (kcal mol^-1^) | FNfn10^b^ $\text{DD}G_{\text{D-N}}$ (kcal mol^-1^) | TNfn3 $\text{DD}G_{\text{D-N}}$ (kcal mol^-1^) |
| --- | --- | --- | --- | --- |
| P5A | A | 0.21 ± 0.20 | 0.11 ± 0.18 | 2.74 ± 0.14 |
| I8A | A | 1.49 ± 0.18 |  | 2.86 ± 0.11 |
| L8A | A |  | 1.64 ± 0.17 |  |
| I20V | B | 0.83 ± 0.19 | 0.41 ± 0.18 | 0.23 ± 0.13 |
| I20A | B | 3.74 ± 0.16 | 0.74 ± 0.17 | 3.67 ±0.11 |
| P25A | B | 0.10 ± 0.20 | 0.48 ± 0.18 | 1.73 ± 0.13 |
| I32A | C | 2.43 ± 0.18 |  | 3.28 ± 0.12 |
| Y32A | C |  | 4.38 ± 0.15 |  |
| L34A | C | 3.45 ± 0.25 |  | 4.29 ± 0.11 |
| I34A | C |  | 5.02 ± 0.15 |  |
| Y36F | C | 0.27 ± 0.20 | 0.67 ± 0.17 | 0.44 ± 0.13 |
| Y36A | C | 2.65 ± 0.17 | 2.29 ± 0.21 | 4.22 ± 0.10 |
| E38A | C | 0.57 ± 0.19 |  |  |
| I48A | C’ | 1.73 ± 0.18 |  | 2.19 ± 0.12 |
| F48A | C’ |  | 2.16 ± 0.16 |  |
| L50A | C’ | 2.99 ± 0.18 |  | 2.99 ± 0.12 |
| V50A | C’ |  | 2.42 ± 0.16 |  |
| Y57A | E | 1.34 ± 0.19 |  | 1.38 ±0.13 |
| A57G | E |  | 2.81 ± 0.16 |  |
| I59A | E | 2.90 ± 0.17 | 3.66 ± 0.15 | 2.06 ± 0.11 |
| L62A | E | 4.56 ± 0.16 | 2.94 ± 0.17 | 4.22 ± 0.11 |
| T66A | E | 1.70 ± 0.18 |  | 1.96 ± 0.12 |
| V66A | E |  | 0.67 ± 0.17 |  |
| Y68F | F | 2.87 ± 0.17 | 2.03 ± 0.16 | 3.13 ± 0.12 |
| Y68A | F | Unfolded | 3.92 ± 0.15 | Unfolded |
| V70A | F | 1.60 ± 0.19 |  | 2.81 ± 0.11 |
| I70A | F |  | 3.72 ± 0.15 |  |
| L72A | F | 2.15 ± 0.19 |  | 3.06 ± 0.13 |
| V72A | F |  | 3.33 ± 0.15 |  |
| S74A | F | -0.61 ± 0.21 |  | -0.04 ± 0.13 |
| A74G | F |  | 1.38 ± 0.17 |  |
| S85A | G | -0.16 ± 0.20 | -0.11 ± 0.18 |  |
| S81A | G |  |  | 2.82 ± 0.11 |
| F92A | G | 4.06 ± 0.17 | 1.30 ± 0.25 |  |
| F88A | G |  |  | 5.43 ± 0.26 |

^a^ Numbering of proteins is the same between positions 1 and 78. There is a four-residue insertion in the F-G loop of FNoTNc and FNfn10, resulting in the numbering of equivalent residues in the G-strand being four numbers lower in TNfn3.

^b^ FNfn10 and TNfn3 data taken from^1^.

1. Cota, E., Hamill, S. J., Fowler, S. B. & Clarke, J. (2000). Two proteins with the same structure respond very differently to mutation: the role of plasticity in protein stability. J. Mol. Biol. 302, 713-725.

**Table S2.** Rate constants for hydrogen exchange of backbone amide protons in FNoTNc

| Residue | H-bond location (donor-receptor) | *k*_ex_ (min^-1^) | (kcal mol^-1^) |
| --- | --- | --- | --- |
| 6 | β_A_-β_B_ | b | b |
| 8 | β_A_-s.chain (90) | a | a |
| 9 | β_A_-β_B_ | 1.5 x 10^-2^ | 4.5 |
| 11 | β_A_-β_B_ | 1.8 x 10^-2^ | 5.4 |
| 12 | β_A_-β_B_ | a | a |
| 14 | β_A_-β_B_ | 2.9 x 10^-2^ | 4.9 |
| 16 | Turn_A-B_-s.chain (14) | b | b |
| 17 | β_B_-s.chain (14) | 2.1 x 10^-3^ | 7.5 |
| 18 | β_B_-β_E_ | 1.1 x 10^-3^ | 7.5 |
| 19 | β_B_-β_A_ | 9.6 x 10^-4^ | 8.8 |
| 20 | β_B_-β_E_ | 1.6 x 10^-3^ | 5.6 |
| 21 | β_B_-β_A_ | 9.6 x 10^-4^ | 8.8 |
| 22 | β_B_-β_E_ | 2.9 x 10^-3^ | 7.8 |
| 23 | β_B_-β_A_ | b | b |
| 30 | Turn_B-C_-β_F_ | 1.6 x 10^-4^ | 8.1 |
| 31 | c | b | b |
| 32 | β_C_-β_C’_ | 4.5 x 10^-3^ | 5.3 |
| 33 | β_C_-β_F_ | 2.9 x 10^-3^ | 6.3 |
| 34 | β_C_-β_C’_ | 1.3 x 10^-3^ | 6.5 |
| 35 | β_C_-β_F_ | 1.3 x 10^-3^ | 6.6 |
| 36 | β_C_-β_C’_ | 1.4 x 10^-2^ | 5.5 |
| 37 | β_C_-β_F_ | 3.5 x 10^-2^ | 5.5 |
| 38 | β_C_-β_C’_ | b | b |
| 39 | Turn_C-C’_-β_F_ | b | b |
| 41 | Turn_C-C’_-β_F_ | a | a |
| 46 | β_C’_-β_C_ | b | b |
| 48 | β_C’_-β_C_ | 3.7 x 10^-3^ | 5.2 |
| 50 | β_C’_-β_C_ | 1.7 x 10^-2^ | 5.0 |
| 53 | Turn_C’-E_- turn_C’-E_ | b | b |
| 56 | c | b | b |
| 57 | β_E_-β_B_ | 1.6 x 10^-3^ | 6.8 |
| 58 | c | b | b |
| 59 | β_E_-β_B_ | 9.1 x 10^-4^ | 6.5 |
| 62 | Turn_E-F_-turn_A-B_ | 7.7 x 10^-3^ | 5.4 |
| 63 | Turn_E-F-_ s.chain (68) | 8.7 x 10^-2^ | 4.2 |
| 65 | β_F_-β_G_ | 9.3 x 10^-2^ | 4.5 |
| 66 | β_F_-s.chain (94) | 7.4 x 10^-4^ | 7.5 |
| 67 | c | b | b |
| 68 | β_F_-β_G_ | 1.6 x 10^-3^ | 6.3 |
| 69 | β_F_-β_C_ | 1.1 x 10^-3^ | 7.1 |
| 70 | β_F_-β_G_ | 1.3 x 10^-3^ | 6.3 |
| 71 | β_F_-β_C_ | 2.5 x 10^-3^ | 6.3 |
| 72 | β_F_-β_G_ | 3.1 x 10^-3^ | 6.0 |
| 73 | β_F_-β_C_ | 5.3 x 10^-3^ | 5.5 |
| 74 | β_F_-s.chain (85) | b | b |
| 75 | β_F_-β_C_ | b | b |
| 76 | β_F_-β_G_ | b | b |
| 79 | β_G_-β_F_ | b | b |
| 85 | β_G_-β_F_ | b | b |
| 88 | β_G_-β_F_ | b | b |
| 90 | β_G_-β_F_ | 1.8 x 10^-3^ | 6.2 |
| 92 | β_G_-β_F_ | 5.4 x 10^-3^ | 6.3 |
| 94 | β_G_-β_F_ | 8.7 x 10^-3^ | 6.1 |
| 95 | β_G_-β_F_ | 7.3 x 10^-2^ | 4.2 |
| 96 | β_G_-β_F_ | 5.9 x 10^-2^ | 1.1 |

^a^ The proteins have different conformations, and different H-bonding partners in these regions.

^b^ Rate constant for exchange too high to be determined.

^c^ The H-bonding partner could not be determined.

**Table S3.** Backbone ^15^N relaxation rates *R*_1_ and *R*_2_ and nOe enhancements for FNoTNc

| Residue | *R*_1_ (s^-1^) | *R*_2_ (s^-1^) | nOe |
| --- | --- | --- | --- |
| 4 | 1.99 (0.02) | 7.56 (0.11) | 0.45 (0.01) |
| 6 | 2.09 (0.03) | 8.01 (0.14) | 0.59 (0.02) |
| 7 | 2.13 (0.03) | 8.36 (0.12) | 0.64 (0.02) |
| 8 | 2.01 (0.01) | 7.65 (0.05) | 0.68 (0.02) |
| 9 | 2.19 (0.02) | 8.20 (0.08) | 0.70 (0.02) |
| 10 | 2.02 (0.03) | 8.02 (0.08) | 0.73 (0.02) |
| 11 | 2.11 (0.03) | 8.75 (0.12) | 0.74 (0.02) |
| 12 | 2.01 (0.01) | 12.73 (0.07) | 0.73 (0.02) |
| 13 | 2.00 (0.01) | 9.78 (0.13) | 0.72 (0.02) |
| 14 | 2.01 (0.02) | 10.85 (0.08) | 0.77 (0.02) |
| 17 | 2.13 (0.01) | 9.59 (0.17) | 0.79 (0.02) |
| 18 | 2.03 (0.01) | 9.60 (0.12) | 0.68 (0.02) |
| 19 | 1.98 (0.01) | 8.58 (0.07) | 0.71 (0.02) |
| 21 | 2.16 (0.02) | 8.89 (0.09) | 0.75 (0.02) |
| 22 | 2.11 (0.03) | 8.98 (0.04) | 0.73 (0.02) |
| 24 | 1.94 (0.01) | 8.14 (0.14) | 0.66 (0.02) |
| 26 | 1.99 (0.03) | 7.66 (0.14) | 0.58 (0.02) |
| 27 | 1.81 (0.06) | 8.52 (0.21) | 0.61 (0.02) |
| 28 | 1.93 (0.06) | 9.60 (0.15) | 0.65 (0.02) |
| 29 | 2.07 (0.03) | 9.21 (0.14) | 0.67 (0.02) |
| 30 | 2.20 (0.05) | 10.52 (0.10) | 0.73 (0.02) |
| 31 | 2.08 (0.02) | 9.60 (0.06) | 0.74 (0.02) |
| 32 | 2.14 (0.01) | 8.94 (0.09) | 0.76 (0.02) |
| 33 | 2.08 (0.02) | 8.55 (0.21) | 0.76 (0.02) |
| 34 | 2.15 (0.02) | 8.58 (0.09) | 0.76 (0.02) |
| 35 | 2.16 (0.02) | 8.89 (0.12) | 0.76 (0.02) |
| 36 | 2.17 (0.02) | 8.73 (0.05) | 0.75 (0.02) |
| 37 | 2.13 (0.02) | 8.66 (0.15) | 0.75 (0.02) |
| 38 | 2.10 (0.01) | 8.22 (0.06) | 0.70 (0.02) |
| 39 | 2.04 (0.01) | 8.73 (0.09) | 0.72 (0.02) |
| 41 | 1.84 (0.04) | 6.63 (0.33) | 0.60 (0.02) |
| 43 | 1.67 (0.03) | 6.40 (0.11) | 0.55 (0.01) |
| 46 | 2.02 (0.02) | 9.17 (0.11) | 0.69 (0.02) |
| 48 | 2.10 (0.01) | 8.05 (0.16) | 0.73 (0.02) |
| 49 | 2.02 (0.01) | 8.18 (0.05) | 0.71 (0.02) |
| 50 | 2.09 (0.01) | 8.30 (0.23) | 0.77 (0.02) |
| 52 | 2.06 (0.03) | 8.83 (0.20) | 0.75 (0.02) |
| 53 | 2.14 (0.04) | 13.22 (0.46) | 0.73 (0.02) |
| 54 | 2.16 (0.02) | 8.82 (0.20) | 0.73 (0.02) |
| 56 | 2.10 (0.01) | 8.96 (0.23) | 0.80 (0.02) |
| 57 | 2.08 (0.01) | 9.19 (0.11) | 0.77 (0.02) |
| 58 | 2.01 (0.02) | 8.01 (0.09) | 0.72 (0.02) |
| 59 | 2.10 (0.02) | 8.48 (0.12) | 0.73 (0.02) |
| 60 | 1.97 (0.01) | 8.84 (0.12) | 0.73 (0.02) |
| 61 | 2.09 (0.09) | 8.34 (0.11) | 0.74 (0.02) |
| 62 | 2.15 (0.01) | 8.57 (0.07) | 0.71 (0.02) |
| 63 | 1.98 (0.02) | 8.53 (0.11) | 0.74 (0.02) |
| 65 | 2.15 (0.01) | 8.58 (0.07) | 0.75 (0.02) |
| 66 | 2.07 (0.01) | 8.40 (0.11) | 0.73 (0.02) |
| 67 | 2.03 (0.01) | 8.40 (0.07) | 0.78 (0.02) |
| 68 | 2.14 (0.02) | 8.19 (0.15) | 0.75 (0.02) |
| 69 | 2.09 (0.02) | 8.36 (0.09) | 0.73 (0.02) |
| 70 | 2.22 (0.02) | 8.35 (0.19) | 0.74 (0.02) |
| 71 | 2.10 (0.01) | 8.52 (0.03) | 0.75 (0.02) |
| 73 | 2.09 (0.03) | 11.69 (0.09) | 0.74 (0.02) |
| 74 | 2.14 (0.03) | 8.69 (0.03) | 0.74 (0.02) |
| 75 | 2.13 (0.02) | 8.28 (0.20) | 0.71 (0.02) |
| 86 | 1.87 (0.02) | 8.02 (0.20) | 0.55 (0.01) |
| 88 | 2.04 (0.02) | 7.47 (0.10) | 0.65 (0.02) |
| 89 | 1.97 (0.02) | 7.70 (0.06) | 0.69 (0.02) |
| 90 | 2.14 (0.01) | 8.62 (0.17) | 0.77 (0.02) |
| 91 | 2.02 (0.02) | 8.14 (0.04) | 0.74 (0.02) |
| 92 | 2.12 (0.02) | 8.55 (0.12) | 0.74 (0.02) |
| 93 | 2.02 (0.02) | 8.10 (0.11) | 0.74 (0.02) |
| 94 | 2.19 (0.02) | 8.80 (0.10) | 0.74 (0.02) |
| 95 | 2.04 (0.02) | 8.73 (0.25) | 0.75 (0.02) |
| 96 | 1.98 (0.02) | 7.62 (0.07) | 0.69 (0.02) |

Errors (± 1 S.D.) are given in parentheses

**Table S4.** Model-free parameters and exchange contributions fitted to backbone ^15^N relaxation data for FNoTNc

| Residue | *S*^2^ | τ_e_ (s) | *R*_ex_  (s^-1^) | | *S*^2^_f_ | |
| --- | --- | --- | --- | --- | --- | --- |
| 4 | 0.77 (0.01) | 1.34e-10 (0.07e-10) |  |  |  |  |
| 6 | 0.83 (0.01) | 1.07e-10 (0.12e-10) |  |  |  |  |
| 7 | 0.86 (0.01) | 1.00e -10 (0.13e-10) |  |  |  |  |
| 8 | 0.92 (0.01) | 9.26e-10 (2.18e-10) |  |  | 0.85 (0.01) | |
| 9 | 0.91 (0.02) | 1.46e-09 (0.41e-09) |  |  | 0.91 (0.01) | |
| 10 | 0.83 (0.01) | 2.75e-11 (0.84e-11) |  |  |  |  |
| 11 | 0.87 (0.01) | 3.05e-11 (1.34e-11) | 0.40 (0.19) | |  |  |
| 12 | 0.83 (0.01) | 2.40e-11 (0.87e-11) | 4.79 (0.09) | |  |  |
| 13 | 0.82 (0.01) | 2.85e-11 (0.86e-11) | 1.87 (0.13) | |  |  |
| 14 | 0.84 (0.01) | 0.00 | 2.84 (0.11) | |  |  |
| 17 | 0.89 (0.01) | 0.00 | 1.10 (0.18) | |  |  |
| 18 | 0.83 (0.01) | 4.89e-11 (0.81e-11) | 1.63 (0.12) | |  |  |
| 19 | 0.81 (0.01) | 3.09e-11 (0.82e-11) | 0.77 (0.07) | |  |  |
| 21 | 0.89 (0.01) | 2.90e-11 (1.62e-11) | 0.34 (0.11) | |  |  |
| 22 | 0.87 (0.01) | 3.79e-11 (1.27e-11) | 0.66 (0.12) | |  |  |
| 24 | 0.79 (0.01) | 4.69e-11 (0.62e-11) | 0.55 (0.16) | |  |  |
| 26 | 0.79 (0.01) | 8.30e-11 (0.74e-11) |  |  |  |  |
| 27 | 0.72 (0.02) | 4.68e-11 (0.65e-11) | 1.53 (0.30) | |  |  |
| 28 | 0.78 (0.03) | 5.03e-11 (1.08e-11) | 2.07 | (0.28) |  |  |
| 29 | 0.84 (0.01) | 5.95e-11 (1.09e-11) | 1.10 (0.20) | |  |  |
| 30 | 0.90 (0.02) | 5.13e-11 (2.41e-11) | 1.85 (0.21) | |  |  |
| 31 | 0.86 (0.01) | 2.82e-11 (1.21e-11) | 1.38 (0.10) | |  |  |
| 32 | 0.89 (0.01) | 0.00 | 0.41 (0.11) | |  |  |
| 33 | 0.87 (0.01) | 0.00 |  |  |  |  |
| 34 | 0.90 (0.01) | 0.00 |  |  |  |  |
| 35 | 0.91 (0.01) | 0.00 |  |  |  |  |
| 36 | 0.91 (0.01) | 0.00 |  |  |  |  |
| 37 | 0.89 (0.01) | 0.00 |  |  |  |  |
| 38 | 0.86 (0.01) | 5.09e-11 (1.02e-11) |  |  |  |  |
| 39 | 0.84 (0.01) | 3.19e-11 (0.90e-11) | 0.69 (0.09) | |  |  |
| 41 | 0.73 (0.01) | 5.06e-11 (0.58e-11) |  |  |  |  |
| 43 | 0.66 (0.01) | 4.83e-11 (0.37e-11) |  |  |  |  |
| 46 | 0.82 (0.01) | 4.68e-11 (0.82e-11) | 1.23 (0.14) | |  |  |
| 48 | 0.86 (0.01) | 3.73e-11 (1.11e-11) |  |  |  |  |
| 49 | 0.83 (0.01) | 3.61e-11 (0.95e-11) | 0.21 (0.06) | |  |  |
| 50 | 0.87 (0.01) | 0.00 |  |  |  |  |
| 52 | 0.86 (0.01) | 0.00 | 0.63 (0.23) | |  |  |
| 53 | 0.88 (0.02) | 3.93e-11 (1.88e-11) | 4.75 (0.45) | |  |  |
| 54 | 0.90 (0.01) | 4.34e-11 (1.73e-11) |  |  |  |  |
| 56 | 0.87 (0.01) | 0.00 | 0.60 (0.22) | |  |  |
| 57 | 0.87 (0.01) | 0.00 | 0.90 (0.11) | |  |  |
| 58 | 0.83 (0.01) | 2.94e-11 (0.84e-11) |  |  |  |  |
| 59 | 0.88 (0.01) | 0.00 |  |  |  |  |
| 60 | 0.81 (0.01) | 2.33e-11 (0.71e-11) | 1.06 (0.12) | |  |  |
| 61 | 0.87 (0.01) | 0.00 |  |  |  |  |
| 62 | 0.88 (0.01) | 4.99e-11 (1.29e-11) |  |  |  |  |
| 63 | 0.82 (0.01) | 2.01e-11 (0.92e-11) | 0.70 (0.14) | |  |  |
| 65 | 0.90 (0.01) | 0.00 |  |  |  |  |
| 66 | 0.86 (0.01) | 2.84e-11 (0.95e-11) |  |  |  |  |
| 67 | 0.84 (0.01) | 0.00 | 0.32 (0.09) | |  |  |
| 68 | 0.95 (0.01) | 2.24e-09 (1.89e-09) |  |  | 0.88 (0.01) | |
| 69 | 0.87 (0.01) | 2.91e-11 (1.10e-11) |  |  |  |  |
| 70 | 0.93 (0.02) | 2.24e-09 (1.57e-09) |  |  | 0.91 (0.01) | |
| 71 | 0.88 (0.01) | 0.00 | 0.13 (0.04) | |  |  |
| 73 | 0.86 (0.01) | 2.82e-11 (1.18e-11) | 3.42 (0.15) | |  |  |
| 74 | 0.91 (0.01) | 0.00 |  |  |  |  |
| 75 | 0.87 (0.01) | 4.95e-11 (1.24e-11) |  |  |  |  |
| 86 | 0.74 (0.01) | 6.96e-11 (0.51e-11) | 0.86 (0.22) | |  |  |
| 88 | 0.87 (0.01) | 1.25e-09 (0.20e-09) |  |  | 0.86 (0.01) | |
| 89 | 0.80 (0.01) | 3.96e-11 (0.70e-11) |  |  |  |  |
| 90 | 0.89 (0.01) | 0.00 |  |  |  |  |
| 91 | 0.85 (0.01) | 0.00 |  |  |  |  |
| 92 | 0.88 (0.01) | 0.00 |  |  |  |  |
| 93 | 0.84 (0.01) | 0.00 |  |  |  |  |
| 94 | 0.91 (0.01) | 4.33e-11 (1.75e-11) |  |  |  |  |
| 95 | 0.84 (0.01) | 2.00e-11 (1.04e-11) | 0.63 (0.28) | |  |  |
| 96 | 0.80 (0.01) | 3.90e-11 (0.71e-11) |  |  |  |  |

Errors (± 1 S.D.) are given in parentheses

**Table S5.** Sidechain methyl deuterium relaxation rates *R*_1_ and *R*_2_ for FNoTNc

| Methyl | *R*_1_ (s^-1^) | *R*_2_ (s^-1^) |
| --- | --- | --- |
| I8 δ1 | 15.57 (0.13) | 67.61 (0.05) |
| I8 γ2 ^α^ | 27.17 (0.06) | 89.65 (0.14) |
| V10 γ1 | 18.26 (0.10) | 88.77 (0.13) |
| V10 γ2 | 16.93 (0.11) | 84.05 (0.11) |
| A12 β ^α^ | 26.54 (0.06) | 88.66 (0.10) |
| V13 γ1 | 22.28 (0.04) | 38.71 (0.04) |
| V13 γ2 ^α^ | 22.67 (0.04) | 43.08 (0.04) |
| T16 γ2 ^α^ | 27.28 (0.21) | 103.99 (0.17) |
| A18 β | 47.15 (0.18) | 183.61 (0.21) |
| L19 δ1 | 21.11 (0.13) | 52.06 (0.10) |
| L19 δ2 ^α^ | 19.33 (0.06) | 54.91 (0.08) |
| I20 δ1 | 23.04 (0.06) | 60.06 (0.08) |
| I20 γ2 | 17.65 (0.09) | 71.18 (0.07) |
| A24 β | 25.11 (0.10) | 79.21 (0.07) |
| A26 β | 27.95 (0.06) | 79.29 (0.04) |
| V27 γ2 ^α^ | 26.93 (0.05) | 64.23 (0.09) |
| T28 γ2 | 25.96 (0.08) | 75.28 (0.11) |
| I29 δ1 | 19.59 (0.16) | 60.12 (0.19) |
| I29 γ2 ^α^ | 32.35 (0.11) | 71.90 (0.13) |
| I32 δ1 | 18.51 (0.09) | 41.85 (0.04) |
| I32 γ2 | 39.58 (0.12) | 71.03 (0.12) |
| L34 δ1 | 21.33 (0.19) | 88.44 (0.17) |
| L34 δ2 ^α^ | 17.79 (0.09) | 53.74 (0.07) |
| T35 γ2 ^α^ | 25.08 (0.07) | 95.84 (0.09) |
| V45 γ1 ^α^ | 27.45 (0.04) | 44.69 (0.05) |
| I48 δ1 | 16.04 (0.07) | 33.28 (0.07) |
| I48 γ2 | 23.83 (0.06) | 59.28 (0.06) |
| T49 γ2 ^α^ | 25.39 (0.05) | 73.01 (0.03) |
| T56 γ2 | 31.81 (0.12) | 93.86 (0.08) |
| T58 γ2 | 22.97 (0.13) | 83.73 (0.15) |
| I59 δ1 | 12.69 (0.18) | 87.90 (0.14) |
| I59 γ2 | 36.36 (0.10) | 97.36 (0.08) |
| L62 δ1 | 29.27 (0.16) | 84.34 (0.18) |
| L62 δ2 | 22.59 (0.20) | 95.50 (0.33) |
| T69 γ2 ^α^ | 28.72 (0.10) | 83.59 (0.11) |
| V70 γ1 ^α^ | 29.48 (0.05) | 66.37 (0.06) |
| V70 γ2 | 38.11 (0.11) | 77.22 (0.06) |
| T71 γ2 ^α^ | 30.15 (0.07) | 107.90 (0.12) |
| L72 δ1 | 22.98 (0.13) | 38.11 (0.10) |
| L72 δ2 | 21.44 (0.06) | 36.72 (0.06) |
| V75 γ1 ^α^ | 24.78 (0.17) | 62.69 (0.10) |
| V75 γ2 | 21.95 (0.13) | 85.73 (0.08) |
| A88 β ^α^ | 34.93 (0.10) | 99.22 (0.13) |
| I90 δ1 | 8.70 (0.14) | 51.94 (0.09) |
| I90 γ2 | 42.69 (0.06) | 106.78 (0.16) |
| T94 γ2 | 41.31 (0.27) | 126.82 (0.11) |
| I96 δ1 | 12.12 (0.06) | 31.02 (0.04) |
| I96 γ2 ^α^ | 23.45 (0.04) | 43.60 (0.03) |

Errors (± 1 S.D.) are given in parentheses

^a^ Overlapped residues.

**Table S6.** Fitted axial order parameters, *S*_axis_^2^, and correlation times, *τ*_e_, for FNoTNc

| Methyl | *S*_axis_^2^ | *τ*_e_ (ps) |
| --- | --- | --- |
| I8 δ1 | 0.55 (0.01) | 24.16 (0.35) |
|  V10 γ1 | 0.75 (0.01) | 26.44 (0.25) |
| V10 γ2 | 0.72 (0.01) | 23.06 (0.43) |
| A12 β  ^a^ | 0.66 (0.01) | 50.27 (0.19) |
| V13 γ1 | 0.17 (0.01) | 50.63 (0.16) |
| V13 γ2  ^a^ | 0.22 (0.01) | 50.55 (0.11) |
| T16 γ2  ^a^ | 0.82 (0.01) | 48.31 (0.62) |
| A18 β | 1.45 (0.01) | 91.72 (0.47) |
| L19 δ1 | 0.33 (0.01) | 44.03 (0.49) |
| L19 δ2  ^a^ | 0.38 (0.01) | 37.94 (0.27) |
| I20 δ1 | 0.40 (0.01) | 45.48 (0.19) |
| I20 γ2 | 0.57 (0.01) | 28.89 (0.78) |
| A24 β | 0.58 (0.01) | 48.49 (0.25) |
| A26 β | 0.55 (0.01) | 56.79 (0.27) |
| V27 γ2  ^a^ | 0.40 (0.01) | 57.75 (0.22) |
| T28 γ2 | 0.53 (0.01) | 52.09 (0.28) |
| I29 δ1 | 0.43 (0.01) | 38.18 (0.56) |
| I29 γ2  ^a^ | 0.42 (0.01) | 71.90 (0.67) |
| I32 δ1 | 0.25 (0.01) | 39.37 (0.27) |
| I32 γ2 | 0.32 (0.01) | 94.83 (0.39) |
| L34 δ1 | 0.72 (0.01) | 35.10 (0.51) |
| L34 δ2  ^a^ | 0.38 (0.01) | 34.20 (0.47) |
| T35 γ2  ^a^ | 0.75 (0.01) | 44.20 (0.26) |
| V45 γ1  ^a^ | 0.18 (0.01) | 64.02 (0.08) |
| I48 δ1 | 0.18 (0.01) | 34.72 (0.16) |
| T49 γ2  ^a^ | 0.51 (0.01) | 50.93 (0.14) |
| T56 γ2 | 0.66 (0.01) | 64.46 (0.61) |
| T58 γ2 | 0.65 (0.01) | 41.56 (0.69) |
| I59 δ1 | 0.80 (0.01) | 9.62 (0.47) |
| I59 γ2 | 0.65 (0.01) | 76.96 (0.66) |
| L62 δ1 | 0.59 (0.01) | 59.40 (0.40) |
| L62 δ2 | 0.78 (0.01) | 35.55 (0.63) |
| T69 γ2  ^a^ | 0.58 (0.01) | 57.91 (0.22) |
| V70 γ2 | 0.42 (0.01) | 86.42 (0.27) |
| T71 γ2  ^a^ | 0.83 (0.01) | 56.38 (0.51) |
| L72 δ1 | 0.16 (0.01) | 52.75 (0.53) |
| V75 γ1  ^a^ | 0.40 (0.01) | 51.80 (0.35) |
| V75 γ2 | 0.68 (0.01) | 37.62 (0.33) |
| A88 β  ^a^ | 0.68 (0.01) | 72.71 (0.26) |
| I90 δ1 | 0.46 (0.01) | 8.83 (0.65) |
| I90 γ2 | 0.68 (0.01) | 93.14 (0.17) |
| T94 γ2 | 0.91 (0.01) | 85.14 (0.97) |
| I96 γ2  ^a^ | 0.21 (0.01) | 52.69 (0.13) |

Errors (± 1 S.D.) are given in parentheses

^a^ Overlapped residues.

**Table S7.** FNoTNc chemical shift assignment table

| Residue | HN shift (ppm) | N shift (ppm) | CO shift (ppm) | CA shift (ppm) | CB shift (ppm) | CG(1,2) shift (ppm) | CD(1,2) shift (ppm) |
| --- | --- | --- | --- | --- | --- | --- | --- |
| S2 | - | - | 172.11 | 58.32 | 64.01 | - | - |
| D3 | 8.361 | 22.14 | 173.35 | 54.22 | 40.96 | - | - |
| V4 | 7.705 | 118.30 | 172.09 | 59.65 | 33.40 | 21.39, 20.42 | - |
| P5 | - | - | 173.24 | 63.22 | 32.15 | 27.94 | 49.22 |
| R6 | 8.450 | 119.67 | 172.84 | 55.22 | 33.01 | 28.02 | 43.55 |
| D7 | 8.583 | 119.22 | 172.73 | 55.06 | 40.53 | - | - |
| I8 | 7.860 | 118.10 | 173.19 | 61.46 | 39.02 | 28.02, 18.13 | 14.84 |
| E9 | 9.366 | 125.96 | 172.55 | 55.10 | 33.78 | 35.91 | - |
| V10 | 8.625 | 123.15 | 174.01 | 61.65 | 31.01 | 21.44, 21.28 | - |
| V11 | 8.919 | 128.69 | 174.03 | 64.01 | 33.09 | 21.38, 21.76 | - |
| A12 | 7.432 | 118.34 | 173.23 | 51.91 | 21.65 | - | - |
| V13 | 8.468 | 117.80 | 172.38 | 61.00 | 35.35 | 22.22, 21.09 | - |
| T14 | 8.736 | 118.40 | 172.27 | 60.55 | 68.70 | 20.62 | - |
| T16 | - | - | 172.66 | 58.96 | 72.04 | 20.82 | - |
| S17 | 7.575 | 116.99 | 169.07 | 56.97 | 68.06 | - | - |
| A18 | 8.395 | 118.27 | 171.83 | 51.75 | 23.58 | - | - |
| L19 | 8.493 | 124.84 | 172.37 | 54.60 | 43.49 | 28.07 | 23.71, 25.43 |
| I20 | 8.922 | 125.48 | 172.43 | 58.05 | 39.49 | 27.22, 18.00 | 12.46 |
| S21 | 9.028 | 118.49 | 170.16 | 56.46 | 66.42 | - | - |
| W22 | 7.921 | 120.28 | 171.65 | 56.90 | 31.01 | - | - |
| D23 | 8.661 | 118.05 | 173.87 | 53.42 | 42.45 | - | - |
| A24 | 8.446 | 126.82 | 173.62 | 50.68 | 18.44 | - | - |
| P25 | - | - | 174.05 | 62.58 | 32.40 | 27.48 | - |
| A26 | 8.398 | 123.16 | 176.24 | 52.88 | 19.32 | - | - |
| V27 | 7.700 | 114.02 | 173.54 | 61.72 | 32.98 | 21.16, 19.58 | - |
| T28 | 7.731 | 113.55 | 172.52 | 62.86 | 69.00 | 22.34 | - |
| I29 | 7.998 | 122.61 | 173.32 | 60.18 | 38.59 | 26.89, 18.40 | 13.28 |
| R30 | 8.634 | 124.77 | 174.77 | 58.15 | 31.49 | 27.38 | 43.22 |
| Y31 | 7.797 | 113.44 | 171.08 | 56.50 | 41.46 | - | - |
| I32 | 9.055 | 119.61 | 172.54 | 58.51 | 40.56 | 27.38, 17.32 | 12.64 |
| R33 | 8.933 | 126.59 | 171.18 | 54.40 | 32.49 | 27.38 | 43.22 |
| L34 | 9.061 | 129.56 | 173.68 | 53.85 | 45.10 | 27.40 | 27.31, 25.57 |
| T35 | 9.475 | 116.63 | 171.84 | 58.82 | 71.63 | 21.22 | - |
| Y36 | 8.617 | 115.82 | 171.63 | 56.09 | 42.36 | - | - |
| G37 | 8.248 | 105.20 | 169.95 | 44.92 | - | - | - |
| E38 | 8.972 | 122.02 | 176.20 | 57.32 | 29.80 | 36.97 | - |
| T39 | 8.583 | 124.58 | 173.85 | 65.06 | 68.88 | - | - |
| S40 | - | - | 172.99 | 45.47 | - | - | - |
| G41 | 7.835 | 106.40 | 172.43 | 45.82 | - | - | - |
| N42 | - | - | 172.91 | 53.44 | 38.96 | - | - |
| S43 | 7.757 | 115.11 | 170.06 | 56.98 | 63.63 | - | - |
| P44 | - | - | 175.20 | 63.24 | 32.17 | 27.53 | 50.89 |
| V45 | 8.376 | 120.15 | 174.25 | 61.82 | 32.98 | 21.42, 20.04 | - |
| Q46 | 8.176 | 122.80 | 172.32 | 54.64 | 30.55 | 34.19 | - |
| E47 | 8.355 | 120.18 | 172.99 | 54.90 | 33.22 | 36.50 | - |
| I48 | 9.386 | 125.76 | 172.38 | 59.51 | 41.34 | 27.48, 17.35 | 13.21 |
| T49 | 8.428 | 121.29 | 171.79 | 62.08 | 69.84 | 21.78 | - |
| L50 | 9.509 | 128.05 | 171.86 | 51.76 | 44.30 | - | 26.46, 23.98 |
| P51 | - | - | 175.61 | 62.99 | 32.40 | 27.86 | 50.53 |
| G52 | 8.081 | 106.98 | 171.07 | 46.59 | - | - | - |
| S53 | 7.390 | 109.32 | 173.11 | 57.84 | 63.51 | - | - |
| K54 | 7.820 | 124.09 | 173.84 | 56.13 | 33.81 | - | - |
| S55 | - | - | 169.23 | 57.26 | 64.65 | - | - |
| T56 | 6.662 | 108.03 | 171.42 | 59.04 | 72.78 | 21.37 | - |
| Y57 | 8.723 | 119.64 | 171.38 | 59.73 | 42.81 | - | - |
| T58 | 7.192 | 123.75 | 170.75 | 61.69 | 69.02 | 20.88 | - |
| I59 | 8.887 | 127.43 | 172.78 | 60.99 | 38.78 | 27.27,18.37 | 14.74 |
| S60 | 8.291 | 120.49 | 171.65 | 57.01 | 65.81 | - | - |
| G61 | 8.636 | 108.04 | 173.36 | 46.55 | - | - | - |
| L62 | 8.315 | 118.52 | 173.09 | 53.38 | 41.45 | - | 24.65, 19.62 |
| K63 | 8.437 | 120.15 | 173.06 | 53.76 | 34.17 | - | - |
| P64 | - | - | 177.51 | 63.40 | 33.55 | 27.38 | 51.42 |
| G65 | 7.970 | 115.33 | 172.26 | 47.92 | - | - | - |
| T66 | 8.354 | 115.89 | 169.11 | 62.99 | 71.63 | 20.08 | - |
| D67 | 8.118 | 125.53 | 173.15 | 54.50 | 42.10 | - | - |
| Y68 | 9.130 | 123.66 | 172.90 | 58.83 | 43.87 | - | - |
| T69 | 8.673 | 114.45 | 172.16 | 61.94 | 72.36 | 21.17 | - |
| V70 | 9.224 | 128.51 | 171.99 | 59.78 | 34.77 | 21.14, 22.97 | - |
| T71 | 9.076 | 122.23 | 170.20 | 61.20 | 70.65 | 21.95 | - |
| L72 | 8.939 | 125.64 | 173.29 | 54.00 | 46.37 | 27.79 | 25.37, 24.67 |
| Y73 | 8.894 | 118.83 | 173.04 | 56.29 | 40.86 | - | - |
| S74 | 8.884 | 117.85 | 171.88 | 58.01 | 64.93 | - | - |
| V75 | 8.477 | 123.45 | 174.07 | 62.02 | 33.36 | 21.63, 20.75 | - |
| T76 | 8.664 | 117.70 | 172.92 | 61.77 | 70.73 | - | - |
| D80 | - | - | 174.15 | 54.32 | 41.19 | - | - |
| S81 | 8.159 | 115.62 | - | 56.37 | 63.57 | - | - |
| P82 | - | - | 174.81 | 63.35 | 32.17 | 27.32 | 50.66 |
| A83 | 8.365 | 123.66 | 175.54 | 52.77 | 19.65 | - | - |
| S85 | - | - | 172.06 | 58.27 | 65.27 | - | - |
| K86 | 8.420 | 122.35 | 172.50 | 54.88 | 32.37 | - | - |
| P87 | - | - | 174.65 | 62.53 | 32.58 | 26.99 | 50.53 |
| A88 | 8.159 | 124.23 | 173.81 | 51.40 | 21.31 | - | - |
| S89 | 8.386 | 114.72 | 171.47 | 57.01 | 66.98 | - | - |
| I90 | 9.019 | 120.65 | 169.79 | 60.36 | 42.80 | 28.76, 17.63 | 15.46 |
| N91 | 8.090 | 121.28 | 172.32 | 51.65 | 41.42 | - | - |
| F92 | 8.792 | 116.64 | 169.04 | 56.91 | 41.48 | - | - |
| R93 | 8.505 | 122.00 | 175.49 | 53.83 | 33.71 | 26.84 | 43.89 |
| T94 | 8.867 | 115.97 | 174.25 | 61.75 | 69.82 | 26.11 | - |
| E95 | 6.448 | 118.65 | 172.66 | 56.38 | 31.96 | 36.78 | - |
| I96 | 7.610 | 121.25 | 178.69 | 63.73 | 40.06 | 18.11 | 14.23 |
