# Supplementary material for: Crosstalk between the Protein Surface and Hydrophobic Core in a Core-swapped Fibronectin Type III Domain
Source: J Mol Biol. 2008 Jan 11;375(2):560–71. doi: 10.1016/j.jmb.2007.10.056 (PMC2291452; doi:10.1016/j.jmb.2007.10.056)
Supplement: Supplementary Tables [file applic2.doc]

**
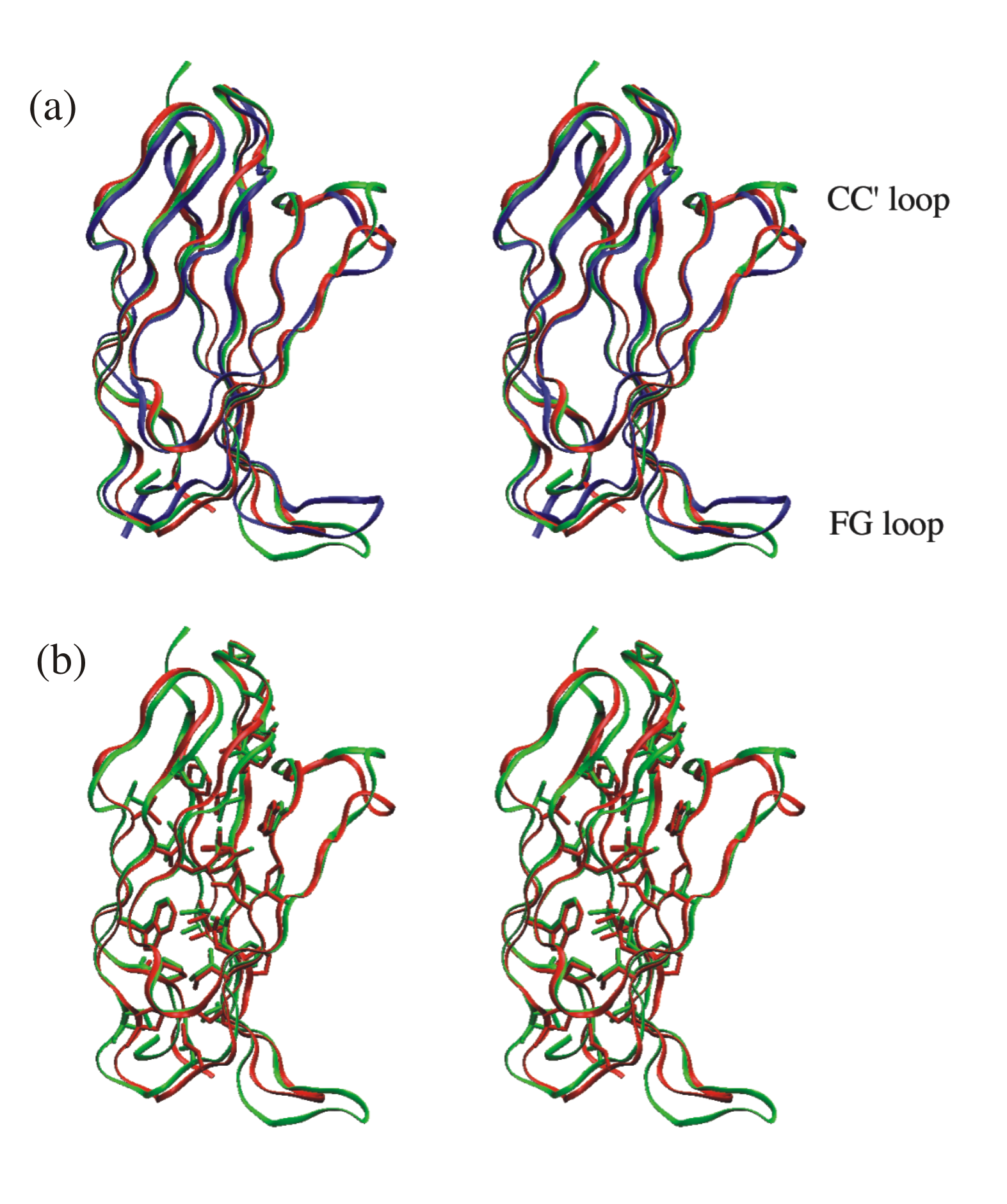
**

(c)

1 10 20 30 40 50

**FNoTNc** VSDVPRDI**EVVAVT**PT**SALISWD**APAVTIR**YIRLTYGE**TGGNSPV**QEITLP**

**FNfn10** VSDVPRDL**EVVAAT**PT**SLLISWD**APAVTVR**YYRITYGE**TGGNSPV**QEFTVP**

**TNfn3** RLDAPSQI**EVKDVT**DT**TALITWF**KPLAEID**GIELTYGI**KDVPGDR**TTIDLT**

***--A--- ---B--- ---C---- --C’--***

52 60 70 80 90

**FNoTNc** GSK**STYTIS**GLKPG**TDYTVTLYSVT**GRGDSPASSKP**ASINFRT**EI

**FNfn10** GSK**STATIS**GLKPG**VDYTITVYAVT**GRGDSPASSKP**ISINYRT**

**TNfn3** EDE**NQYSIG**NLKPD**TEYEVSLISRR**G....DMSSNP**AKETFTT**GL

***--E--- -----F----- ---G---***

**Supplementary Figure S1.** FNoTNc retains the structure of its parents. (a) Stereo view showing an overlay of the backbone traces of FNoTNc (green), FNfn10 (blue) and TNfn3 (red). The backbone RMSD with FNfn10 and TNfn3 is 0.95 Å and 0.89 Å respectively, excluding the C-C’ and F-G loops*,* which have been shown to be flexible in FNfn10 by backbone dynamics experiments^1; 2^. It has also been shown that the FG loop differs by more than 6.0 Å RMS between two crystal structures of FNfn10^3; 4^. (b) Stereo view showing an overlay of FNoTNc (green) and TNfn3 (red). The core residues have the same conformation. (c) Structure-based sequence alignment of FNoTNc, FNfn10 and TNfn3. The conserved core residues are shaded, the swapped core residues in FNoTNc are underlined. The strands are represented with bold letters and are labelled below the sequence. Figures taken from Ng et al.^5^.

**References for Figure S1**

1. Carr, P. A., Erickson, H. P. & Palmer, A. G., 3rd. (1997). Backbone dynamics of homologous fibronectin type III cell adhesion domains from fibronectin and tenascin. *Structure* **5**, 949-959.

2. Meekhof, A. E., Hamill, S. J., Arcus, V. L., Clarke, J. & Freund, S. M. (1998). The dependence of chemical exchange on boundary selection in a fibronectin type III domain from human tenascin. *J. Mol. Biol.* **282**, 181-194.

3. Dickinson, C. D., Veerapandian, B., Dai, X. P., Hamlin, R. C., Xuong, N. H., Ruoslahti, E. & Ely, K. R. (1994). Crystal structure of the tenth type III cell adhesion module of human fibronectin. *J. Mol. Biol.* **236**, 1079-1092.

4. Leahy, D. J., Aukhil, I. & Erickson, H. P. (1996). 2.0 A crystal structure of a four-domain segment of human fibronectin encompassing the RGD loop and synergy region. *Cell* **84**, 155-164.

5. Ng, S. P., Billings, K. S., Ohashi, T., Allen, M. D., Best, R. B., Randles, L. G., Erickson, H. P. & Clarke, J. (2007). Designing an extracellular matrix protein with enhanced mechanical stability. *Proc. Natl. Acad. Sci. USA* **104**, 9633-9637.


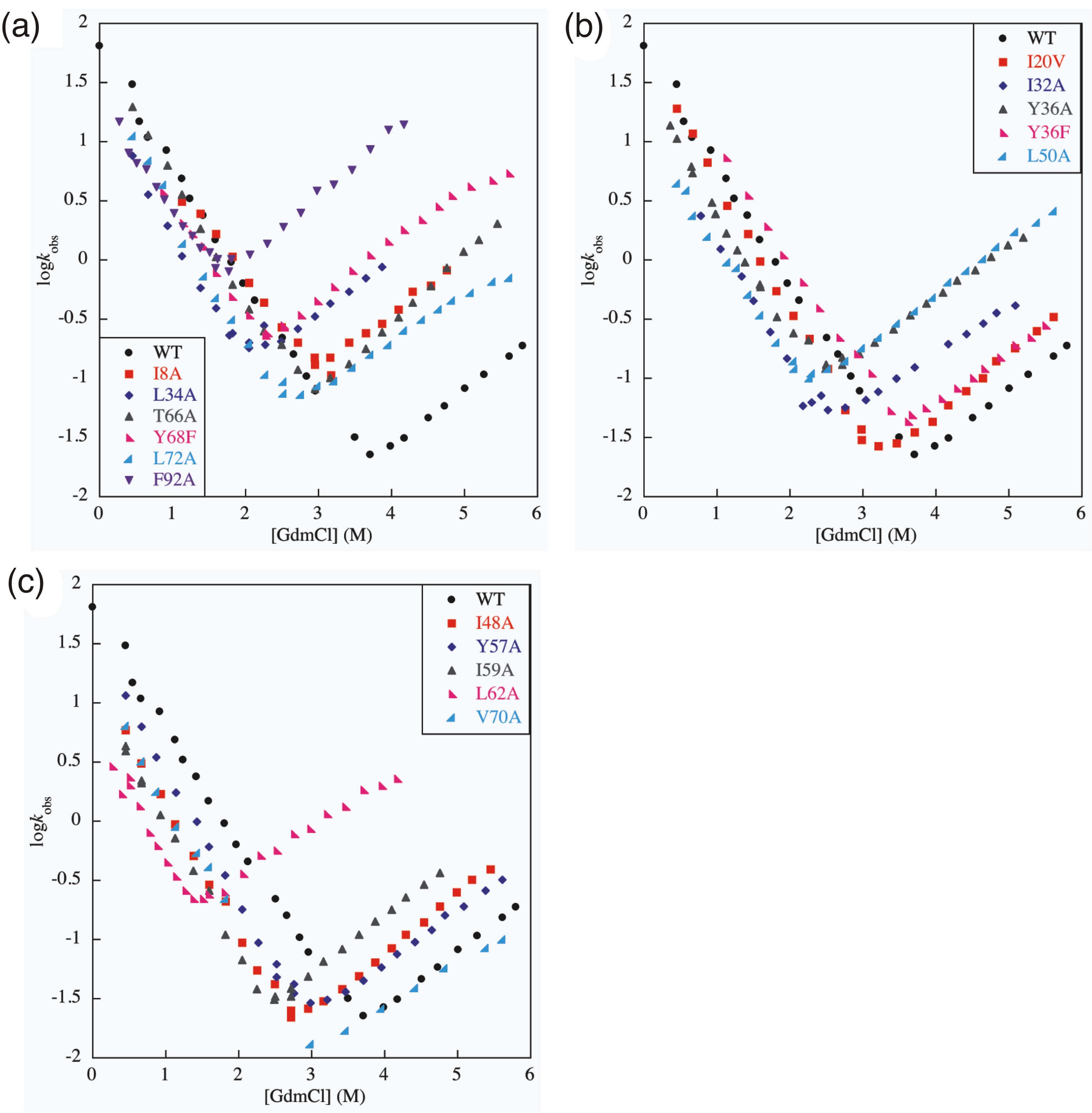


**Supplementary Figure S2.** Folding kinetics of FNoTNc and mutants. Chevron plots for mutants with (a) Low Φ-values < 0.25. (b) 0.25 < Medium Φ-values < 0.35. (c) High Φ-values > 0.35.


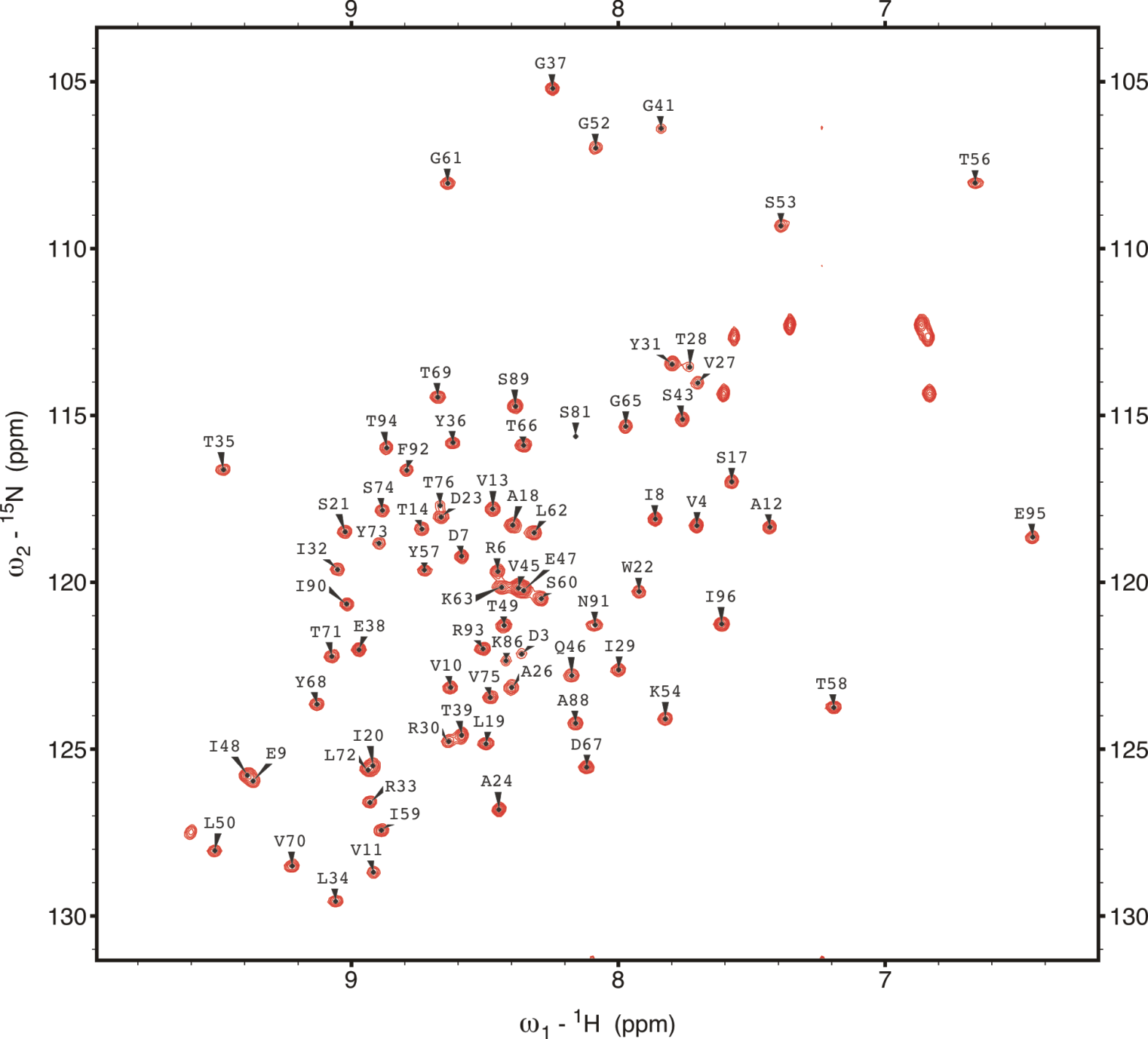


**Supplementary Figure S3.** HSQC of FNoTNc showing assignments


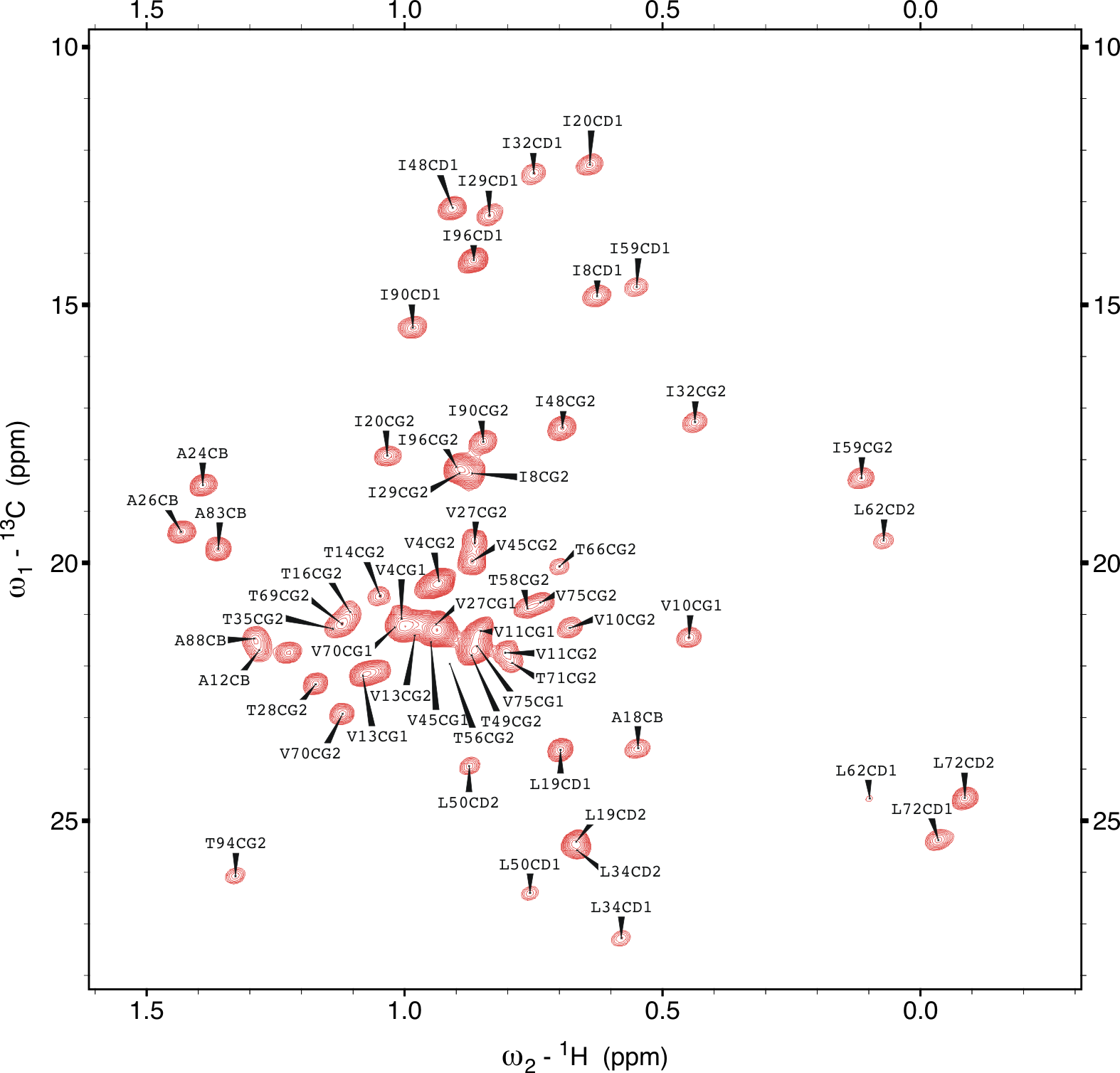


**Supplementary Figure S4.** Sidechain methyl assignments of FNoTNc
